# Offline and online coupled tensor factorization with knowledge graph

**DOI:** 10.1371/journal.pone.0336100

**Published:** 2025-11-12

**Authors:** SeungJoo Lee, Yong-Chan Park, U. Kang

**Affiliations:** 1 IPAI, Seoul National University, Seoul, Republic of Korea; 2 Department of CSE, Seoul National University, Seoul, Republic of Korea; Public Library of Science, UNITED KINGDOM OF GREAT BRITAIN AND NORTHERN IRELAND

## Abstract

How can we accurately decompose a temporal irregular tensor along while incorporating a related knowledge graph tensor in both offline and online streaming settings? PARAFAC2 decomposition is widely applied to the analysis of irregular tensors consisting of matrices with varying row sizes. In both offline and online streaming scenarios, existing PARAFAC2 methods primarily focus on capturing dynamic features that evolve over time, since data irregularities often arise from temporal variations. However, these methods tend to overlook static features, such as knowledge-based information, which remain unchanged over time.

In this paper, we propose KG-CTF (Knowledge Graph-based Coupled Tensor Factorization) and OKG-CTF (Online Knowledge Graph-based Coupled Tensor Factorization), two coupled tensor factorization methods designed to effectively capture both dynamic and static features within an irregular tensor in offline and online streaming settings, respectively. To integrate knowledge graph tensors as static features, KG-CTF and OKG-CTF couple an irregular temporal tensor with a knowledge graph tensor by sharing a common axis. Additionally, both methods employ relational regularization to preserve the structural dependencies among the factor matrices of the knowledge graph tensor. To further enhance convergence speed, we utilize momentum-based update strategies for factor matrices. Through extensive experiments, we demonstrate that KG-CTF reduces error rates by up to 1.64× compared to existing PARAFAC2 methods. Furthermore, OKG-CTF achieves up to 5.7× faster running times compared to existing streaming approaches for each newly arriving tensor.

## Introduction

Given a temporal irregular tensor and a knowledge graph tensor, how can we accurately decompose these tensors to derive meaningful latent factors? Many real-world datasets are represented as irregular tensors such as stock data, traffic data, and music data. An irregular tensor consists of matrices with varying row sizes, while the column sizes remain fixed. For example, in stock data, each matrix corresponds to a specific stock, where the number of rows varies based on different time periods, but the columns represent common stock features such as opening price, closing price, and trading volume.

Large-scale factual information is effectively organized into a knowledge graph, which represents information through entities and the relationships between them. A knowledge graph is structured as a graph-based database that represents information in the form of triples (*e*_*s*_, *r*, *e*_*o*_), where the subject (head) entity *e*_*s*_ is connected to the object (tail) entity *e*_*o*_ through the relation *r*. A knowledge graph can be modeled as a third-order binary array in tensor form, where each entry represents a triplet, with a value of 1 indicating a known fact and 0 representing an unknown or missing fact. Various tensor decomposition methods for knowledge graphs [1–5] have been developed to capture hidden relational patterns and improve the accuracy of missing information prediction.

Many tensor decomposition methods [6–12] have been proposed to extract patterns from high-dimensional data. Recently, there has been much attention on PARAFAC2 decomposition [13–17] in order to analyze irregular tensors. These approaches [14,16] primarily employ the Alternating Least Squares (ALS) algorithm, incorporating various optimization techniques to enhance the efficiency of updates. However, many existing PARAFAC2 decomposition methods have significant limitations. First, they emphasize modeling temporal dynamic features (e.g., daily stock prices and trading volumes), which leads to neglecting static features (e.g., a company’s sector) that remain unchanged over time in the tensor. Both dynamic and static features are equally crucial for the accurate modeling of factor matrices. Second, incorporating static features into the existing PARAFAC2 decomposition method [14,18] often introduces a significant bias toward the temporal features, ultimately failing to explicitly model auxiliary information. TASTE [19] incorporates both dynamic and static features through coupled matrix and tensor factorization; however, it fails to represent static features as high-dimensional data, potentially resulting in information loss. Third, while existing PARAFAC2-based decomposition methods [15,20] introduce additional regularization terms to incorporate information from multiple sources, these terms often hinder convergence during the learning process. Therefore, the major challenges to be addressed are: 1) how to effectively integrate dynamic and static information in irregular tensors, 2) how to accurately capture patterns within static information, and 3) how to develop an update rule for stable convergence.

In this paper, we propose KG-CTF and OKG-CTF, two accurate coupled tensor factorization methods to integrate dynamic and static information in irregular tensors by leveraging knowledge graphs. KG-CTF runs in an offline setting, while OKG-CTF is designed for an online streaming setting. The main ideas of KG-CTF and OKG-CTF are as follows: 1) integrate a temporal irregular tensor with a knowledge graph tensor to model both dynamic and static features, 2) introduce an effective regularization that captures the relationships within the factor matrices of the knowledge graph, and then 3) ensure stable and fast convergence of the complex loss function through a momentum-based ALS update mechanism. Furthermore, in an online streaming setting, OKG-CTF efficiently deals with newly incoming tensors by avoiding direct computations on the entire tensor, instead leveraging only the new data and updated factor matrices. Experimental results show that KG-CTF and OKG-CTF outperform existing PARAFAC2 decomposition methods in missing value prediction tasks, achieving superior accuracy in both offline and online settings.

The contributions of this paper are summarized as follows:

- **Method.** We propose KG-CTF and OKG-CTF, accurate tensor factorization methods that couple knowledge graphs with temporal irregular tensors in both offline and online streaming settings.
- **Theory.** We provide a theoretical analysis of the convergence of KG-CTF and OKG-CTF, demonstrating that they effectively decrease the loss function.
- **Experiments.** Extensive experiments demonstrate that KG-CTF and OKG-CTF reduce error rates by up to 1.64× and improve running times by up to 5.7× compared to existing PARAFAC2 methods. Additionally, we open-source a large-scale knowledge graph containing stock information from South Korea, the United States, Japan, and China.

The code and datasets are available at https://github.com/snudatalab/KG-CTF.

## Preliminaries and problem definition

In this section, we explain preliminaries of irregular tensor notations, PARAFAC2 decomposition, and knowledge graph, followed by the problem definitions. Table 1 summarizes the symbols used throughout the paper.

**Table 1:** Symbol description.

Symbol	Description
$𝒯={\left\{{𝐓}_{k}\right\}}_{k=1}^{K}$	Irregular tensor of slices ${𝐓}_{k}$ for k=1,...,K
$𝒢={\left\{{𝐆}_{k}\right\}}_{k=1}^{K}$	Knowledge graph tensor of slices ${𝐆}_{k}$ for k=1,...,K
${𝐓}_{k,old}$	Old rows of *k*-th existing slice matrix
${𝐓}_{k,new}$	New rows of *k*-th existing slice matrix
${𝐓}_{k}$	*k*-th slice matrix from an irregular tensor
${𝐆}_{k}$	*k*-th slice matrix from a knowledge graph tensor
${𝐔}_{k},{𝐐}_{k},{𝐒}_{k}$	Factor matrices of the *k*-th slice for irregular tensor
𝐇,𝐕	Factor matrices of an irregular tensor
${𝐌}_{k}$	Factor matrix of the *k*-th slice for knowledge graph tensor
R	Factor matrix for relations of knowledge graph tensor
$1_{H}$	All-ones matrix
1	All-ones vector
${𝐃}_{k}$	Degree matrix of relations in the *k*-th slice
*R*	Target rank
vec(·)	Vectorization of a matrix
*	Element-wise product
⊙	Khatri-Rao product
$\|\cdot {\|}_{F}$	Frobenius norm

### Irregular tensor

An irregular tensor is denoted as $𝒳=\{{𝐗}_{k}{\}}_{k=1}^{K}$, consisting of a collection of slice matrices, where each slice ${𝐗}_{k}\in {\mathbb{R}}^{I_{k}\times J}$ represents the *k*-th matrix, and *K* is the total number of slices. Note that the row size *I*_*k*_ varies across slice matrices, while the column size *J* remains the same for all.

### PARAFAC2 decomposition

**Fig 1:**
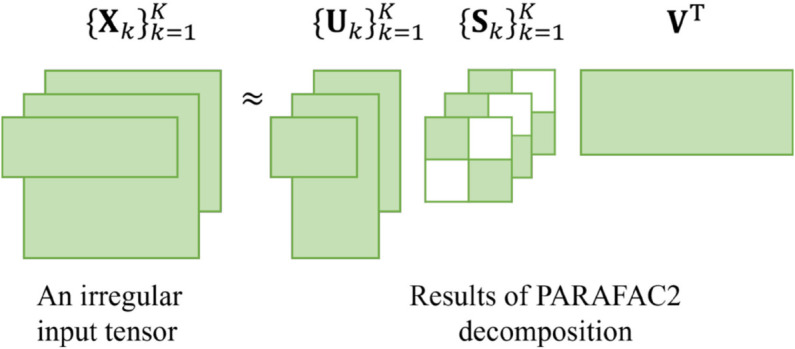
An example of PARAFAC2 decomposition. PARAFAC2 converts each slice matrix ${𝐗}_{k}$ into ${𝐔}_{k}\in {\mathbb{R}}^{I_{k}\times R}$, ${𝐒}_{k}\in {\mathbb{R}}^{R\times R}$, and $𝐕\in {\mathbb{R}}^{J\times R}$ for k=1,...,K, where ${𝐒}_{k}$ is a diagonal matrix.

PARAFAC2 decomposition [21] has been widely used for analyzing irregular tensors. Given each *k*-th slice matrix ${𝐗}_{k}\in {\mathbb{R}}^{I_{k}\times J}$ of an irregular tensor $𝒳=\{{𝐗}_{k}{\}}_{k=1}^{K}$, PARAFAC2 decomposes each slice into three matrices: ${𝐔}_{k}$, ${𝐒}_{k}$, and **V**, as illustrated in Fig 1. This decomposition is formulated as ${𝐗}_{k}\approx {𝐔}_{k}{𝐒}_{k}{𝐕}^{T}$, where ${𝐔}_{k}$ is an $I_{k}\times R$ matrix, ${𝐒}_{k}$ is an R×R diagonal matrix, and **V** is a J×R matrix shared across all slices with *R* as the target rank.

The objective function of the PARAFAC2 decomposition is formulated as follows:


$${ℒ}_{\text{PARAFAC2}}=\sum_{k=1}^{K}\|{𝐗}_{k}-{𝐔}_{k}{𝐒}_{k}{𝐕}^{T}{\|}_{F}^{2}$$

where $\|𝐗{\|}_{F}$ represents the Frobenius norm of matrix **X**, defined as $\|𝐗{\|}_{F}=\sqrt{\sum_{i,j}(𝐗(i,j){)}^{2}}$, with 𝐗(i,j) denoting the (*i*,*j*)-th element of matrix **X**.

To ensure the uniqueness of the solution, several studies [22,23] reformulate ${𝐔}_{k}$ with ${𝐐}_{k}𝐇$, where ${𝐐}_{k}\in {\mathbb{R}}^{I_{k}\times R}$ is a column-orthogonal matrix, and $𝐇\in {\mathbb{R}}^{R\times R}$ is a shared matrix across all slices.

### Knowledge graph

A knowledge graph is a collection of triplets that encode facts through entities and their relationships, denoted as 𝐆={(h,r,t)}. Specifically, each triplet (*h*,*r*,*t*) captures the semantic connection between a head entity *h* and a tail entity *t* through the relationship *r*. For example, the triplet (Harry Potter, Written by, J.K. Rowling) represents the relationship between the book Harry Potter (head entity) and the author J.K. Rowling (tail entity) through the relation Written by in the context of book knowledge. Similarly, in the context of travel, (Central Park, Located in, New York City) indicates that Central Park (head entity) is related to New York City (tail entity) via the relation Located in. This structured representation of a knowledge graph facilitates the efficient integration of large-scale factual information, serving as additional contextual data. A knowledge graph is expressed as a three-dimensional tensor 𝒢, which takes the form of either a regular or an irregular tensor. In this paper, we represent the knowledge graph 𝒢 as an irregular tensor ${\left\{{𝐆}_{k}\right\}}_{k=1}^{K}$, where each ${𝐆}_{k}$ is an item-specific slice matrix that forms an (entity-relation) matrix, with rows corresponding to item-related entities and columns representing relations. A more detailed explanation of this representation is provided later in this paper.

### Problem definition

We define the problems of coupled tensor factorization with knowledge graph in offline and online streaming settings as follows:

**Problem 1** (Coupled Tensor Factorization with Knowledge Graph).

**Given.**

*(1) A temporal irregular tensor* $𝒯={\left\{{𝐓}_{k}\right\}}_{k=1}^{K}$ *with slice matrices* ${𝐓}_{k}\in {\mathbb{R}}^{I_{k}\times J}$
*(2) A knowledge graph tensor* $𝒢={\left\{{𝐆}_{k}\right\}}_{k=1}^{K}$ *with slice matrices* ${𝐆}_{k}\in {\mathbb{R}}^{N_{k}\times L}$

**Find.** Factor matrices ${𝐔}_{k}\in {\mathbb{R}}^{I_{k}\times R}$, ${𝐒}_{k}\in {\mathbb{R}}^{R\times R}$, ${𝐌}_{k}\in {\mathbb{R}}^{N_{k}\times R}$, and ${𝐐}_{k}\in {\mathbb{R}}^{I_{k}\times R}$ for k=1,...,K, and common factor matrices $𝐕\in {\mathbb{R}}^{J\times R}$, $𝐑\in {\mathbb{R}}^{L\times R}$, and $𝐇\in {\mathbb{R}}^{R\times R}$ with *R* as the target rank, where each *k*-th slice matrix of the temporal irregular tensor ${𝐓}_{k}$ is approximated by ${𝐔}_{k}{𝐒}_{k}{𝐕}^{T}$, each *k*-th slice matrix of the knowledge graph tensor ${𝐆}_{k}$ by ${𝐌}_{k}{𝐒}_{k}{𝐑}^{T}$, and each temporal factor matrix ${𝐔}_{k}$ by ${𝐐}_{k}𝐇$.

**Problem 2** (Online Coupled Tensor Factorization with Knowledge Graph). **Given.**

*(1) New rows* $\{{𝐓}_{k,\text{new}}{\}}_{k=1}^{K}$ *of existing slice matrices in a streaming temporal irregular tensor* $𝒯={\left\{{𝐓}_{k}\right\}}_{k=1}^{K}$
*(2) A knowledge graph tensor* $𝒢={\left\{{𝐆}_{k}\right\}}_{k=1}^{K}$ *with slice matrices* ${𝐆}_{k}\in {\mathbb{R}}^{N_{k}\times L}$
*(3) Pre-existing factor matrices* $\{{𝐔}_{k,old}{\}}_{k=1}^{K}$, $\{{𝐐}_{k,old}{\}}_{k=1}^{K}$, $\{{𝐒}_{k}{\}}_{k=1}^{K}$, $\{{𝐌}_{k}{\}}_{k=1}^{K}$, **V**, **H***, and* **R** *of accumulated irregular tensors* $\{{𝐓}_{k,\text{old}}{\}}_{k=1}^{K}$, $\{{𝐆}_{k}{\}}_{k=1}^{K}$

**Find.** *Factor matrices* ${𝐔}_{k}\in {\mathbb{R}}^{I_{k}\times R}$, ${𝐒}_{k}\in {\mathbb{R}}^{R\times R}$, ${𝐌}_{k}\in {\mathbb{R}}^{N_{k}\times R}$*, and* ${𝐐}_{k}\in {\mathbb{R}}^{I_{k}\times R}$ *for* k=1,...,K*, and common factor matrices* $𝐕\in {\mathbb{R}}^{J\times R}$, $𝐑\in {\mathbb{R}}^{L\times R}$*, and* $𝐇\in {\mathbb{R}}^{R\times R}$ *for the entire tensor* $\{{𝐓}_{k}{\}}_{k=1}^{K}$ *and* $\{{𝐆}_{k}{\}}_{k=1}^{K}$


$${𝐓}_{k}=[\begin{array}{c} {𝐓}_{k,\text{old}};{𝐓}_{k,\text{new}} \end{array}],\;\text{for }k=1,\ldots ,K$$

$${𝐔}_{k}=[\begin{array}{c} {𝐔}_{k,\text{old}};{𝐔}_{k,\text{new}} \end{array}],\;\text{for }k=1,\ldots ,K$$

$${𝐐}_{k}=[\begin{array}{c} {𝐐}_{k,\text{old}};{𝐐}_{k,\text{new}} \end{array}],\;\text{for }k=1,\ldots ,K$$

*where; denotes the vertical concatenation of matrices, old rows of k-th existing slice matrix* ${𝐓}_{k,old}$ *are approximated by* ${𝐔}_{k,old}{𝐒}_{k}{𝐕}^{T}$*, and new rows of k-th existing slice matrix* ${𝐓}_{k,new}$ *are approximated by* ${𝐔}_{k,new}{𝐒}_{k}{𝐕}^{T}$*. Each temporal factor matrix* ${𝐔}_{k,old}$ *is approximated by* ${𝐐}_{k,old}𝐇$, ${𝐔}_{k,new}$ *by* ${𝐐}_{k,new}𝐇$*, and each k-th slice matrix of the knowledge graph tensor* ${𝐆}_{k}$ *by* ${𝐌}_{k}{𝐒}_{k}{𝐑}^{T}$.

## Proposed method for offline tensors: KG-CTF

We propose KG-CTF (Knowledge Graph-based Coupled Tensor Factorization), an accurate coupled tensor factorization method designed to effectively capture both dynamic and static features in irregular tensors.

### Overview

KG-CTF tackles the following challenges to achieve accurate coupled tensor factorization.

C1. **Incorporating the static features in data.** While dynamic features effectively capture temporal variations, they often fail to represent time-invariant characteristics inherent in the data. How can we effectively integrate both dynamic and static information for irregular tensor data?
C2. **Capturing the relational patterns of knowledge graphs.** Knowledge graphs are structured in a triple format, where two entities are connected through a specific relationship. Effectively learning these relational dependencies is essential for leveraging static information. How can we accurately capture the relational structures of knowledge graphs?
C3. **Accelerating ALS updates.** Traditional ALS-based PARAFAC2 decomposition methods involve multiple regularization terms, often resulting in slow and unstable convergence. How can we accelerate ALS updates while optimizing complex loss functions?

**Fig 2:**
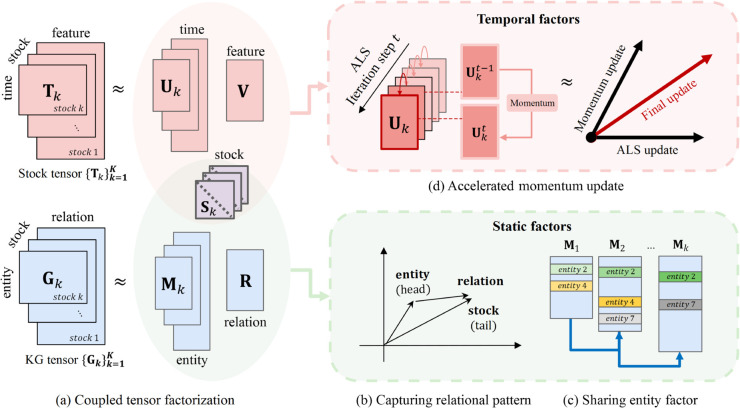
Overview of KG-CTF. (a) We introduce a method that couples a temporal tensor with a Knowledge Graph (KG) tensor by sharing the diagonal matrix *S*_*k*_. This coupling allows the factor matrices to jointly learn both temporal and static features within an irregular tensor. (b) Relational regularization captures the relationship between the factor matrices derived from the KG tensor, improving their interpretability. (c) To ensure consistent entity representations across all slices, we initialize the entity factor matrix ${𝐌}_{k}$ with the updated entity embeddings from ${𝐌}_{1},\cdots ,{𝐌}_{k-1}$. This approach maintains coherence in entity factors across slices, promoting stable learning. (d) We apply a momentum update strategy to ensure faster and stable convergence for factor matrices. At each iteration *t*, the momentum update refines the current ALS update step for ${𝐔}_{k}^{t}$ by incorporating the update direction from ${𝐔}_{k}^{t-1}$, leading to a more efficient final update step for ${𝐔}_{k}^{t}$.

To address these challenges, we propose the following key ideas (see Fig 2):

I1. **Formulating a loss function for PARAFAC2-based coupled tensor factorization with a knowledge graph** integrates temporal irregular tensors with knowledge graph tensors, effectively incorporating both dynamic and static information.
I2. **Relational regularization** enhances the learning of complex relational dependencies among factor matrices representing entities and relationships in the knowledge graph, thereby improving the representation of static information.
I3. **Momentum-based ALS** utilizes past update directions to accelerate ALS-based factor matrix updates, ensuring faster and more stable convergence.

### Coupled tensor factorization

We formulate a loss function that effectively learns both static and dynamic information during the decomposition of irregular tensors. To capture dynamic information, we leverage the temporally irregular tensor $\{{𝐓}_{k}{\}}_{k=1}^{K}$. As described in Eq (1), each slice matrix ${𝐓}_{k}\in {\mathbb{R}}^{I_{k}\times J}$ is factorized using the PARAFAC2 decomposition with a target rank *R*, yielding three factor matrices: ${𝐔}_{k}\in {\mathbb{R}}^{I_{k}\times R}$, ${𝐒}_{k}\in {\mathbb{R}}^{R\times R}$, and $𝐕\in {\mathbb{R}}^{J\times R}$. For example, in stock data analysis, ${𝐔}_{k}$ represents the time factor matrix, ${𝐒}_{k}$ corresponds to the stock-specific factor matrix, and **V** serves as the shared stock feature matrix. The loss function ${ℒ}_{temporal}$ designed to capture this dynamic information is formulated as follows:

$${ℒ}_{temporal}=\sum_{k=1}^{K}{\left\|{𝐓}_{k}-{𝐔}_{k}{𝐒}_{k}{𝐕}^{T}\right\|}_{F}^{2}$$ (1)

Next, we introduce the knowledge graph tensor as a source of static information and integrate it with the existing temporally irregular tensor $\{{𝐓}_{k}{\}}_{k=1}^{K}$. To effectively model the knowledge graph, we represent it as an irregular tensor for two key reasons. First, the number of entities associated with each item varies, inherently resulting in an irregular tensor structure. Second, the conventional entity-relation-entity representation of knowledge graphs leads to exceedingly large and sparse entity factor matrices due to the vast number of entities involved. If the entity factor matrix was constructed using the complete set of entities, the resulting tensor would be highly sparse, making an irregular tensor representation a more effective alternative. To construct this irregular tensor for knowledge graph, we define a slice matrix ${𝐆}_{k}$ for each item *k*, where rows correspond to entities linked to *k*-th item and columns represent all relation types. Specifically, each matrix ${𝐆}_{k}$ is derived from knowledge graph triplets (*h*,*r*,*t*), where the tail entity *t* denotes the *k*-th item, the head entity *h* is an entity associated with that item, and *r* is the relation type. The (*i*,*j*)-th entry of ${𝐆}_{k}$ is set to 1 if the *i*-th entity is connected to item *k* via the *j*-th relation, and 0 otherwise. For example, in a stock-related knowledge graph, each item corresponds to a specific stock, with ${𝐆}_{k}$ representing its entity–relation matrix for each stock. Triplets such as (California, located in, Apple) and (IT, industry of, Apple) indicate that the stock Apple is linked to entities California and IT via the relations located in and industry of, respectively. Accordingly, in ${𝐆}_{k}$ for Apple, the row for California has a 1 in the located in column, and the row for IT has a 1 in the industry of column. Therefore, we define the knowledge graph tensor as $\{{𝐆}_{k}{\}}_{k=1}^{K}$, where each slice matrix ${𝐆}_{k}\in {\mathbb{R}}^{N_{k}\times L}$ (representing entity-relation interactions for an item) is factorized into three components: ${𝐌}_{k}\in {\mathbb{R}}^{N_{k}\times R}$, ${𝐒}_{k}\in {\mathbb{R}}^{R\times R}$, and $𝐑\in {\mathbb{R}}^{L\times R}$. Here, ${𝐌}_{k}$ is the entity factor matrix, ${𝐒}_{k}$ is the item-specific factor matrix, and **R** is the relation factor matrix. In this notation, *N*_*k*_ denotes the number of entities linked to the *k*-th item, *L* represents the total number of relation types, and *R* is the target rank. For stock datasets, ${𝐆}_{k}$ is decomposed into ${𝐌}_{k}$, ${𝐒}_{k}$, and **R**, representing entity-specific features related to each stock, stock-level latent factors, and the relation factor matrix shared across all stocks, respectively. Therefore, the loss function ${ℒ}_{static}$ for the learning of static information is formulated as follows:


$${ℒ}_{static}=\sum_{k=1}^{K}{\left\|{𝐆}_{k}-{𝐌}_{k}{𝐒}_{k}{𝐑}^{T}\right\|}_{F}^{2}$$

To ensure consistent entity representations across different slices and mitigate the inconsistencies that arise from independently decomposing each slice in PARAFAC2, we introduce a shared entity factor matrix approach. As shown in Fig 2(c), the embedding of an entity remains the same across all slices, preserving its representation throughout the decomposition process. For each slice *k*, the entity factor matrix ${𝐌}_{k}$ is initialized using the following strategy: (1) If an entity has appeared in previous slices, its embedding is inherited from the corresponding entity factors in ${𝐌}_{1},\cdots ,{𝐌}_{k-1}$, (2) otherwise, its embedding is randomly initialized. This iterative approach ensures the stability of entity representations while also enhancing their interpretability across slices.

Finally, we couple the knowledge graph tensor with the temporal irregular tensor by sharing the common axis ${𝐒}_{k}$. Since ${𝐒}_{k}$ is a diagonal matrix that encodes slice-specific information, sharing it allows for simultaneous learning of the dynamic characteristics from the temporal tensor and the static properties embedded in the knowledge graph tensor. Furthermore, unlike other factor matrices (e.g., ${𝐔}_{k}$, **V**, ${𝐌}_{k}$, or **R**) that are domain-specific, ${𝐒}_{k}$ represents item-level latent factors. Therefore, sharing this static matrix ${𝐒}_{k}$, which is unaffected by temporal dynamics, ensures interpretability while avoiding conflicts between modalities. The overall loss function ${ℒ}_{CTF}$ that couples these two tensors is formulated as follows:


$${ℒ}_{CTF}=\;{ℒ}_{temporal}+{ℒ}_{static}\;=\;\sum_{k=1}^{K}\left({\left\|{𝐓}_{k}-{𝐔}_{k}{𝐒}_{k}{𝐕}^{T}\right\|}_{F}^{2}+{\left\|{𝐆}_{k}-{𝐌}_{k}{𝐒}_{k}{𝐑}^{T}\right\|}_{F}^{2}\right)$$

### Regularization

We add effective regularizations to the loss function.

#### Relational regularization.

We introduce a relational regularization term to ensure that the factor matrices obtained from the knowledge graph tensor effectively encode relational structures. Existing methods [24–26] struggle to fully capture the intricate relational dependencies present in knowledge graphs, largely because they overlook the directional nature of these relationships. To address this shortcoming, we incorporate a regularization term that explicitly models the triple structure of the knowledge graph, thereby improving the representation of directional relationships.

Based on the approach proposed in [27], we assume that each knowledge graph triplet (*h*,*r*,*t*) satisfies the condition ‖h+r−t‖≈0, where *h*, *r*, and *t* correspond to the vector representations of the head entity, relation, and tail entity, respectively. Here, the norm represents the Euclidean distance, enforcing relational consistency within the knowledge graph. For instance, in a stock-related knowledge graph, if the head entity is iPhone, the relation is produced by, and the tail entity is Apple Inc., the relationship is captured by ensuring that ‖iPhone+producedby−AppleInc.‖ remains close to zero.

To effectively learn these relational dependencies, we decompose each knowledge graph slice ${𝐆}_{k}\in {\mathbb{R}}^{N_{k}\times L}$ into three factor matrices. The entity factor matrix ${𝐌}_{k}\in {\mathbb{R}}^{N_{k}\times R}$ represents the head entities, the relation factor matrix $𝐑\in {\mathbb{R}}^{L\times R}$ captures relational properties, and the item factor matrix ${𝐒}_{k}\in {\mathbb{R}}^{R\times R}$ serves as the representation of the tail entities. To handle cases where an item is connected to multiple entities through the same relation, the regularization term is minimized using the least squares method.

**Fig 3:**
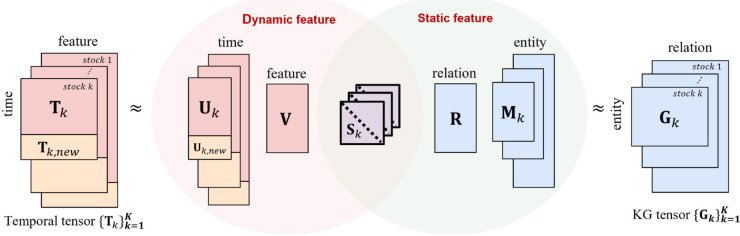
Example of relational regularization for a head stock *h.* Given four relation types and three entities connected to stock *h*, the slice matrix ${𝐆}_{h}\in {\mathbb{R}}^{3\times 4}$ encodes binary connections between entities and relations. The relational regularization term aligns the tail embeddings $1_{H}{𝐒}_{h}\in {\mathbb{R}}^{4\times R}$ of stock *h* with the sum of the relation embeddings $𝐑\in {\mathbb{R}}^{4\times R}$ and the average head embeddings ${𝐃}_{h}^{-1}{𝐆}_{h}^{T}{𝐌}_{h}\in {\mathbb{R}}^{4\times R}$ computed over entities connected to stock *h* per relation.

For instance, if an item *i*_1_ is linked to entities *e*_2_ and *e*_3_ through relation *r*_1_, the objective is to minimize ${\left\|i_{1}-r_{1}-e_{2}\right\|}_{2}^{2}+{\left\|i_{1}-r_{1}-e_{3}\right\|}_{2}^{2}$. Given that *e*_2_ and *e*_3_ are fixed, this can be equivalently rewritten as minimizing ${\left\|i_{1}-r_{1}-\frac{e_{2}+e_{3}}{2}\right\|}_{2}^{2}$. Here, $(e_{2}\;+\;e_{3})/2$ represents the average embedding of *e*_2_ and *e*_3_. Thus, to incorporate this principle, we define the relational regularization term as follows:


$$\lambda_{r}{\left\|1_{H}{𝐒}_{k}-𝐑-{𝐃}_{k}^{-1}{𝐆}_{k}^{T}{𝐌}_{k}\right\|}_{F}^{2}$$

where ${𝐃}_{k}\in {\mathbb{R}}^{L\times L}$ is the diagonal degree matrix associated with ${𝐆}_{k}\in {\mathbb{R}}^{N_{k}\times L}$, and the (*l*,*l*)-th element of ${𝐃}_{k}$ corresponds to the number of entities connected to the *l*-th relation (i.e., the sum of the elements in the *l*-th column of ${𝐆}_{k}$). Additionally, $1_{H}\in {\mathbb{R}}^{L\times R}$ is a matrix filled with ones. $1_{H}{𝐒}_{k}\in {\mathbb{R}}^{L\times R}$ represents the tail, meaning the embedding of the *k*-th item padded *L* times. The matrix $𝐑\in {\mathbb{R}}^{L\times R}$ represents the relation factor, while ${𝐃}_{k}^{-1}{𝐆}_{k}^{T}{𝐌}_{k}\in {\mathbb{R}}^{L\times R}$ represents the head, computed as the average embedding of entities linked by the same relation. Here, ${𝐌}_{k}\in {\mathbb{R}}^{N_{k}\times R}$ represents the embeddings of the *N*_*k*_ entities connected to the *k*-th item. The product ${𝐆}_{k}^{T}{𝐌}_{k}\in {\mathbb{R}}^{L\times R}$ yields a matrix where the *l*-th row contains the sum of embeddings of entities linked to *k*-th item through relation *l*. By applying ${𝐃}_{k}^{-1}$, we normalize this sum by the number of entities per relation, so that the *l*-th row of ${𝐃}_{k}^{-1}{𝐆}_{k}^{T}{𝐌}_{k}$ corresponds to the average embedding of entities connected to *k*-th item via relation *l*. To further illustrate how the relational regularization term operates, we provide an example in Fig 3. This figure illustrates a case where a head stock *h* is connected to three entities via four relation types, resulting in a slice matrix ${𝐆}_{h}\in {\mathbb{R}}^{3\times 4}$. The regularization encourages the tail embedding of stock *h*, repeated four times as $1_{H}{𝐒}_{h}\in {\mathbb{R}}^{4\times R}$, to be aligned with the sum of the relation embeddings $𝐑\in {\mathbb{R}}^{4\times R}$ and the average head entity embeddings associated with each relation, computed as ${𝐃}_{h}^{-1}{𝐆}_{h}^{T}{𝐌}_{h}\in {\mathbb{R}}^{4\times R}$. This alignment reduces inconsistency between the stock embedding and its relational context in the knowledge graph, resulting in more semantically coherent representations across domains.

#### Uniqueness regularization.

To enhance the interpretability of the factor matrices while preserving accuracy, we employ uniqueness regularization by reformulating ${𝐔}_{k}$ as ${𝐐}_{k}𝐇$, as established in previous studies [22,23], where ${𝐐}_{k}$ is a column-orthogonal matrix. This approach mitigates the issue of arbitrary rotations in factor matrices, ensuring that ${𝐐}_{k}𝐇$ remains unique, up to scaling and permutation. To enforce this constraint, we incorporate the following regularization terms into Eq (1):


$$\lambda_{u}{\left\|{𝐔}_{k}-{𝐐}_{k}𝐇\right\|}_{F}^{2}$$

where $\lambda_{u}$ is a hyperparameter to control the effect of uniqueness for ${𝐔}_{k}$.

#### L2 regularization.

We apply L2 regularization to the factor matrices ${𝐒}_{k}$, ${𝐌}_{k}$, **V**, and **R** to mitigate overfitting and enhance numerical stability throughout the optimization process:


$$\lambda_{l}{\left\|{𝐒}_{k}\right\|}_{F}^{2},\;\lambda_{l}{\left\|{𝐌}_{k}\right\|}_{F}^{2},\;\lambda_{l}{\left\|𝐕\right\|}_{F}^{2},\;\lambda_{l}{\left\|𝐑\right\|}_{F}^{2}$$

#### Loss function.

We define the following loss function ${ℒ}_{KG-CTF}$, incorporating effective regularization terms:

$${ℒ}_{KG-CTF}=\;{ℒ}_{temporal}+{ℒ}_{static}+{ℒ}_{reg}\;=\sum_{k=1}^{K}({\left\|{𝐓}_{k}-{𝐔}_{k}{𝐒}_{k}{𝐕}^{T}\right\|}_{F}^{2}+\lambda_{u}{\left\|{𝐔}_{k}-{𝐐}_{k}𝐇\right\|}_{F}^{2}\;+{\left\|{𝐆}_{k}-{𝐌}_{k}{𝐒}_{k}{𝐑}^{T}\right\|}_{F}^{2}+\lambda_{r}{\left\|1_{H}{𝐒}_{k}-𝐑-{𝐃}_{k}^{-1}{𝐆}_{k}^{T}{𝐌}_{k}\right\|}_{F}^{2}\;+\lambda_{l}\left({\left\|{𝐒}_{k}\right\|}_{F}^{2}+{\left\|{𝐌}_{k}\right\|}_{F}^{2}\right))+\lambda_{l}\left({\left\|𝐑\right\|}_{F}^{2}+{\left\|𝐕\right\|}_{F}^{2}\right)$$ (2)

**Algorithm 1 KG-CTF with momentum-based ALS.**

**Input:** $T_{k}\in {\mathbb{R}}^{I_{k}\times J}$ and ${𝐆}_{k}\in {\mathbb{R}}^{N_{k}\times L}$ for k=1,…,K

**Output:** ${𝐔}_{k}\in {\mathbb{R}}^{I_{k}\times R}$, ${𝐐}_{k}\in {\mathbb{R}}^{I_{k}\times R}$, ${𝐒}_{k}\in {\mathbb{R}}^{R\times R}$, ${𝐌}_{k}\in {\mathbb{R}}^{N_{k}\times R}$, for k=1,…,K, $𝐕\in {\mathbb{R}}^{J\times R}$, $𝐇\in {\mathbb{R}}^{R\times R}$, and $𝐑\in {\mathbb{R}}^{L\times R}$

**Parameter:** target rank *R* and momentum strength *β*

1: Initialize matrices ${𝐔}_{k},{𝐐}_{k},{𝐒}_{k},{𝐌}_{k}$ for k=1,…,K, and 𝐕,𝐇,

and **R**

2: **repeat**

3:   **for** k=1,…,K **do**

4:    Update ${𝐔}_{k}$ using Eq (3)

5:    ${𝐔}_{k}\leftarrow {𝐔}_{k}+\beta ({𝐔}_{k}-{𝐔}_{k}^{prev})$, and ${𝐔}_{k}^{prev}\leftarrow {𝐔}_{k}$

6:    Update ${𝐒}_{k}$ using Eq (4)

7:    ${𝐒}_{k}\leftarrow {𝐒}_{k}+\beta ({𝐒}_{k}-{𝐒}_{k}^{prev})$, and ${𝐒}_{k}^{prev}\leftarrow {𝐒}_{k}$

8:    Update ${𝐌}_{k}$ using Eq (8)

9:    Update ${𝐐}_{k}$ using Eq (6)

10:   **end for**

11:   Update **V** using Eq (5)

12:   $𝐕\leftarrow 𝐕+\beta (𝐕-{𝐕}^{prev})$, and ${𝐕}^{prev}\leftarrow 𝐕$

13:   Update **R** using Eq (9)

14:   Update **H** using Eq (7)

15: **until** the maximum iteration is reached, or the error ceases

to decrease

### Momentum update procedure

We introduce a Momentum-based ALS algorithm that leverages a momentum mechanism to enhance the convergence speed of ALS when optimizing complex loss functions. By incorporating a fraction of the prior update direction into the newly computed factor matrix, the algorithm achieves more stable and faster factor updates. For the factor matrix ${𝐔}_{k}$, let ${𝐔}_{k}^{t}$ denote its value at iteration *t*. After performing the standard ALS update to compute ${𝐔}_{k}^{t}$, the update is further refined by incorporating a momentum term:


$${𝐔}_{k}^{t}\leftarrow {𝐔}_{k}^{t}+\beta ({𝐔}_{k}^{t}-{𝐔}_{k}^{t-1})$$

where *β* is the momentum coefficient, typically ranging between 0 and 1. During the initial iterations (e.g., *t* < 5), *β* is set to zero to allow the algorithm to stabilize without the influence of previous updates. In subsequent iterations, the momentum coefficient *β* is assigned a positive value to integrate a portion of the previous update direction into the current update, thus accelerating convergence and enhancing optimization efficiency. By incorporating this momentum mechanism, the ALS algorithm improves its ability to process high-dimensional data, ultimately achieving faster and more stable convergence.

Algorithm 1 presents an overview of the proposed Momentum-ALS algorithm. The algorithm starts with the random initialization of the factor matrices, followed by iterative ALS updates. For each slice *k*, the factor matrices are initially updated using the standard ALS procedure, and a momentum term is subsequently applied to refine the final update.

We use an alternating optimization-based update procedure, where each factor matrix is updated independently while keeping the other factor matrices fixed.

**Updating ${𝐔}_{k}$.** We update ${𝐔}_{k}$ by setting $\frac{\partial ℒ}{\partial {𝐔}_{k}}=0$ and rearranging the terms with the following lemma:

**Lemma 1.** *When fixing all the factor matrices except for* ${𝐔}_{k}$*, the following update for* ${𝐔}_{k}$ *minimizes* ${ℒ}_{KG-CTF}$ *(Eq (*2*)):*

$${𝐔}_{k}\leftarrow \left({𝐓}_{k}𝐕{𝐒}_{k}+\lambda_{u}{𝐐}_{k}𝐇\right)\;\times {\left({𝐒}_{k}{𝐕}^{T}𝐕{𝐒}_{k}+\lambda_{u}𝐈\right)}^{-1}$$ (3)

□

*Proof*: See Appendix. □

**Updating ${𝐒}_{k}$.** To update ${𝐒}_{k}$, we first transform ${𝐒}_{k}$ for *k* = 1...*K* into $𝐖\in {\mathbb{R}}^{K\times R}$ whose *k*-th row contains the diagonal elements of ${𝐒}_{k}$ (i.e., $𝐖(k,r)={𝐒}_{k}(r,r)$). Then, we update 𝐖(k,:) by setting $\frac{\partial ℒ}{\partial 𝐖(k,:)}=0$ and rearranging the term based on the loss function (Eq (2)). We update **W** row by row with the following lemma:

**Lemma 2.** *When fixing all the factor matrices except for* 𝐖*, the following update for the k-th row of the factor matrix* 𝐖 *which corresponds to the diagonal elements of* ${𝐒}_{k}$ *minimizes* ${ℒ}_{KG-CTF}$ *(Eq (*2*)):*

$$𝐖(k,:)\leftarrow (vec({𝐓}_{k}{)}^{T}(𝐕⊙{𝐔}_{k})+vec({𝐆}_{k}{)}^{T}(𝐑⊙{𝐌}_{k})+\lambda_{r}1^{T}(𝐑+{𝐃}_{k}^{-1}{𝐆}_{k}^{T}{𝐌}_{k}))\;\times ({𝐕}^{T}𝐕*{𝐔}_{k}^{T}{𝐔}_{k}+{𝐑}^{T}𝐑*{𝐌}_{k}^{T}{𝐌}_{k}+\lambda_{r}L𝐈+\lambda_{l}𝐈{)}^{-1}$$ (4)

*where* vec(·) *is a vectorization of a matrix.* ⊙ and * denote the Khatri-Rao product and the element-wise multiplication, respectively. $1\in {\mathbb{R}}^{L\times 1}$ *is a vector filled with ones.* □

*Proof*: See Appendix.

**Updating 𝐕.** We update 𝐕 by setting $\frac{\partial ℒ}{\partial 𝐕}=0$ and rearranging the terms with the following lemma:

**Lemma 3.** *When fixing all the factor matrices except for* 𝐕*, the following update for* 𝐕 *minimizes* ${ℒ}_{KG-CTF}$ *(Eq (*2*)):*

$$𝐕\leftarrow \left(\sum_{k=1}^{K}\left({𝐓}_{k}^{T}{𝐔}_{k}{𝐒}_{k}\right)\right)\;\times {\left(\lambda_{l}𝐈+\sum_{k=1}^{K}\left({𝐒}_{k}{𝐔}_{k}^{T}{𝐔}_{k}{𝐒}_{k}\right)\right)}^{-1}$$ (5)

□

*Proof*: See Appendix.

**Updating ${𝐐}_{k}$ and 𝐇.** We update ${𝐐}_{k}$ and 𝐇, which are factor matrices for the uniqueness regularization, with the following lemma:

**Lemma 4.** *When fixing all the factor matrices except for* ${𝐐}_{k}$*, the following update for the matrix* ${𝐐}_{k}$ *minimizes the loss function (Eq (*2*)):*

$${𝐐}_{k}\leftarrow {𝐙}_{k}{𝐏}_{k}^{T}$$ (6)

*where* ${𝐐}_{k}$ *is a column-orthogonal matrix (i.e.,* ${𝐐}_{k}^{T}{𝐐}_{k}=𝐈$)*, and* ${𝐙}_{k}$ *and* ${𝐏}_{k}^{T}$ *are left and right singular vector matrices of* ${𝐔}_{k}{𝐇}^{T}$, *respectively.* □

**Proof**: See Appendix.

**Lemma 5.** *When fixing all the factor matrices except for* 𝐇*, the following update for the factor matrix* 𝐇 *minimizes the loss function (Eq (*2*)):*

$$𝐇\leftarrow \frac{1}{K}\sum_{k=1}^{K}{𝐐}_{k}^{T}{𝐔}_{k}$$ (7)

□

*Proof*: See Appendix.

**Updating ${𝐌}_{k}$.** We update ${𝐌}_{k}$ by setting $\frac{\partial ℒ}{\partial {𝐌}_{k}}=0$ and rearranging the terms with the following lemma:

**Lemma 6.** *Solving the Sylvester equation (Eq (*8*)) with respect to* ${𝐌}_{k}$ *minimizes the loss function (Eq (*2*)) when all other factor matrices are fixed:*

$${𝐌}_{k}{𝐒}_{k}{𝐑}^{T}𝐑{𝐒}_{k}+\left(\lambda_{r}{𝐆}_{k}{\left({𝐃}_{k}^{-1}\right)}^{2}{𝐆}_{k}^{T}+\lambda_{l}𝐈\right){𝐌}_{k}\;={𝐆}_{k}𝐑{𝐒}_{k}+\lambda_{r}{𝐆}_{k}{𝐃}_{k}^{-1}\left(1_{H}{𝐒}_{k}-𝐑\right)$$ (8)

*Note that the Sylvester equation [28] has the form* 𝐀𝐗+𝐗𝐁=𝐂*, where we set* ${𝐌}_{k}=𝐗$ *to solve for* ${𝐌}_{k}$. □

*Proof*: See Appendix. □

**Updating 𝐑.** We update 𝐑 by setting $\frac{\partial ℒ}{\partial 𝐑}=0$ and rearranging the terms with the following lemma:

**Lemma 7.** *When fixing all the factor matrices except for* 𝐑, *the following update for the factor matrix* 𝐑 *minimizes the loss function (Eq (*2*)):*

$$𝐑\leftarrow \left(\sum_{k=1}^{K}\left({𝐆}_{k}^{T}{𝐌}_{k}{𝐒}_{k}+\lambda_{r}\left(1_{H}{𝐒}_{k}-{𝐃}_{k}^{-1}{𝐆}_{k}^{T}{𝐌}_{k}\right)\right)\right)\;\times {\left((\lambda_{l}+\lambda_{r}K)𝐈+\sum_{k=1}^{K}\left({𝐒}_{k}{𝐌}_{k}^{T}{𝐌}_{k}{𝐒}_{k}\right)\right)}^{-1}$$ (9)

□

*Proof*: See Appendix. □

We iteratively update the factor matrices using the update procedure in Lemmas 1 to 7. Each update is the exact closed-form ALS solution of a quadratic subproblem with all other factors fixed, which ensures monotonic decrease of the objective and convergence to a stationary local minimum.

### Time complexity of KG-CTF

We provide the time complexity of KG-CTF.

**Theorem 1.** *The time complexity of KG-CTF is given by*


$$𝒪(KR^{2}(J+L+R)+\sum_{k=1}^{K}(R(J+R)I_{k}+R(L+R)N_{k}+N_{k}^{2}R+N_{k}^{3})).$$

□

*Proof*: See Appendix.

According to Theorem 1, KG-CTF runs in time that scales linearly with the total number $\sum_{k=1}^{K}I_{k}J$ of tensor entries, ensuring practicality even for very large datasets. It is also noteworthy that, in most practical settings, the KG information tables are orders of magnitude smaller than the primary tensor ($N_{k}\ll I_{k}$). Consequently, the cubic term $𝒪(N_{k}^{3}$  +  $N_{k}^{2}R)$ is asymptotically dominated by the linear term in *I*_*k*_, and its contribution to the overall runtime is negligible.

## Proposed method for online tensors: OKG-CTF

We propose OKG-CTF (Online Knowledge Graph-based Coupled Tensor Factorization), an accurate coupled tensor factorization method designed to handle both dynamic and static features in streaming irregular tensor data.

### Overview

OKG-CTF addresses the following challenges for accurate coupled tensor factorization in an online streaming setting.

C1. **Incorporating static features into streaming tensor data.** Existing methods in online streaming settings focus only on dynamic characteristics, ignoring the time-invariant features inherent in the data. How can we efficiently employ both dynamic and static features for streaming irregular tensor data?
C2. **Improving efficiency in an online streaming setting.** Previous coupled tensor factorization methods fail to work efficiently for streaming data due to repeated computations involving old data. How can we minimize the computational cost and space cost as new tensor data arrive over time?

**Fig 4:**
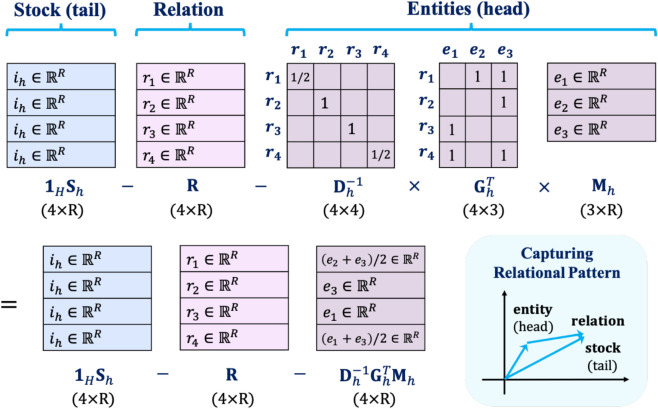
Overview of OKG-CTF. We formulate a method to couple a streaming temporal tensor with a knowledge graph tensor by sharing the diagonal matrix *S*_*k*_. This enables factor matrices to jointly learn temporal and static features within an irregular tensor in an online setting.

We address the above challenges with the following ideas (see Fig 4):

I1. **Formulating a loss function for PARAFAC2-based streaming coupled tensor factorization using a knowledge graph** enables the effective integration of streaming temporal irregular tensors with knowledge graph tensors, capturing both dynamic and static information in an online setting.
I2. **Avoiding explicit computations on old data** enables OKG-CTF to efficiently update factor matrices by dividing the terms related to old and new data.

### Loss function for a streaming setting

We propose a loss function designed to efficiently capture both dynamic and static features from a streaming temporal irregular tensor $\{{𝐓}_{k}{\}}_{k=1}^{K}$. The loss function in Eq (2), when applied in streaming settings, suffers from inefficiencies due to redundant computations of the accumulated tensor whenever new data are given. This highlights the need for a more effective strategy tailored for a streaming setting. Therefore, we reformulate the loss function by separating the terms related to old data from those related to new data. When new data are added to each slice matrix of the temporal irregular tensor, the loss function is defined as follows:

$$L_{\text{OKG-CTF}}=\sum_{k=1}^{K}(\lambda_{f}{\left\|{𝐓}_{k,old}-{𝐔}_{k,old}{𝐒}_{k}{𝐕}^{T}\right\|}_{F}^{2}+{\left\|{𝐓}_{k,new}-{𝐔}_{k,new}{𝐒}_{k}{𝐕}^{T}\right\|}_{F}^{2}\;+{\left\|{𝐆}_{k}-{𝐌}_{k}{𝐒}_{k}{𝐑}^{T}\right\|}_{F}^{2}+\lambda_{r}{\left\|1_{H}{𝐒}_{k}-𝐑-{𝐃}_{k}^{-1}{𝐆}_{k}^{T}{𝐌}_{k}\right\|}_{F}^{2}\;+\lambda_{u}\left(\lambda_{f}{\left\|{𝐔}_{k,old}-{𝐐}_{k,old}𝐇\right\|}_{F}^{2}+{\left\|{𝐔}_{k,new}-{𝐐}_{k,new}𝐇\right\|}_{F}^{2}\right)\;+\lambda_{l}\left({\left\|{𝐒}_{k}\right\|}_{F}^{2}+{\left\|{𝐌}_{k}\right\|}_{F}^{2}\right))+\lambda_{l}\left({\left\|𝐑\right\|}_{F}^{2}+{\left\|𝐕\right\|}_{F}^{2}\right)$$ (10)

where $\lambda_{f}$ ($0<\lambda_{f}\leq 1$) is the hyperparameter controlling the effect of forgetting factor, which determines the relative importance of newly arrived data compared to older data. Specifically, we divide ${𝐓}_{k}\in {\mathbb{R}}^{I_{k}\times J}$ into ${𝐓}_{k,old}\in {\mathbb{R}}^{I_{k,old}\times J}$ and ${𝐓}_{k,new}\in {\mathbb{R}}^{I_{k,new}\times J}$, ${𝐔}_{k}\in {\mathbb{R}}^{I_{k}\times R}$ into ${𝐔}_{k,old}\in {\mathbb{R}}^{I_{k,old}\times R}$ and ${𝐔}_{k,new}\in {\mathbb{R}}^{I_{k,new}\times R}$, and ${𝐐}_{k}\in {\mathbb{R}}^{I_{k}\times R}$ into ${𝐐}_{k,old}\in {\mathbb{R}}^{I_{k,old}\times R}$ and ${𝐐}_{k,new}\in {\mathbb{R}}^{I_{k,new}\times R}$. This approach ensures efficient computation by avoiding redundant operations on old data and focusing on learning from newly arrived data. The forgetting factor $\lambda_{f}$ plays a crucial role in controlling the balance between retaining historical information and adapting to recent changes. A smaller $\lambda_{f}$ emphasizes recent data, enabling rapid adaptation to new patterns but increasing sensitivity to short-term noise. Conversely, a larger $\lambda_{f}$ gives greater weight to historical information, enhancing stability while slowing adaptation to distributional shifts. Thus, $\lambda_{f}$ defines the trade-off between adaptivity and stability, highlighting the importance of selecting an appropriate balanced value in practice.

### Streaming update procedure

We propose an efficient ALS update rule optimized for streaming settings, where one factor matrix is updated independently while fixing the other factor matrices. This rule eliminates the inefficiency caused by repeatedly recalculating terms involving old data whenever new data arrive, focusing on the computation of only new data.

When new data are added, the loss function is divided into two parts: one for the old data and another for the new data. During the update process, terms related to old data are loaded without any recomputation, while only the terms associated with the new data are calculated. In the subsequent step, as additional new data arrive, the previously loaded old factor matrix is combined with the computed factor matrix to form the updated old factor matrix, which is then loaded in the next step. This method effectively avoids unnecessary computational growth as data accumulate, ensuring consistent efficiency over time.

Algorithm 2 provides an overview of the proposed Momentum-ALS algorithm for OKG-CTF in a streaming setting. It starts by randomly initializing new factor matrices, followed by iterative ALS updates. For each slice *k*, all factor matrices are updated using standard ALS, with a momentum term applied to refine the final updates. The updated new factor matrices are then merged with the existing ones to update the old factor matrices for the next streaming data.

**Algorithm 2 OKG-CTF with momentum-based ALS.**

**Input:** a new incoming $\{{𝐓}_{k,new}{\}}_{k=1}^{K}$, $\{{𝐆}_{k}{\}}_{k=1}^{K}$, pre-existing factor

matrices $\{{𝐔}_{k,old}{\}}_{k=1}^{K}$, $\{{𝐐}_{k,old}{\}}_{k=1}^{K}$, $\{{𝐒}_{k}{\}}_{k=1}^{K}$, $\{{𝐌}_{k}{\}}_{k=1}^{K}$, **V**, **H**,

and **R**

**Output:** updated factor matrices $\{{𝐔}_{k,new}{\}}_{k=1}^{K}$, $\{{𝐐}_{k,new}{\}}_{k=1}^{K}$,

$\{{𝐒}_{k}{\}}_{k=1}^{K}$, $\{{𝐌}_{k}{\}}_{k=1}^{K}$, **V**, **H**, and **R**

**Parameter:** target rank *R*, momentum strength *β*

1: Initialize matrices ${𝐔}_{k,new},{𝐐}_{k,new}$ for k=1,…,K

2: **repeat**

3:   **for** k=1,…,K **do**

4:    Update ${𝐔}_{k,new}$ using Eq (11)

5:    ${𝐔}_{k,new}\leftarrow {𝐔}_{k,new}+\beta ({𝐔}_{k,new}-{𝐔}_{k,new}^{prev})$, and ${𝐔}_{k,new}^{prev}\leftarrow {𝐔}_{k,new}$

6:    Update ${𝐒}_{k}\leftarrow 𝐖(k,:)$ using Eq (12)

7:    ${𝐒}_{k}\leftarrow {𝐒}_{k}+\beta ({𝐒}_{k}-{𝐒}_{k}^{prev})$, and ${𝐒}_{k}^{prev}\leftarrow {𝐒}_{k}$

8:    Update ${𝐌}_{k}$ using Eq (8)

9:    Update ${𝐐}_{k,new}$ using Eq (14)

10:   **end for**

11:   Update **V** using Eq (13)

12:   $𝐕\leftarrow 𝐕+\beta (𝐕-{𝐕}^{prev})$, and ${𝐕}^{prev}\leftarrow 𝐕$

13:   Update **R** using Eq (9)

14:   Update **H** using Eq (15)

15: **until** the maximum iteration is reached, or the error ceases

to decrease

16: Update ${𝐔}_{k,old}\leftarrow [{𝐔}_{k,old};{𝐔}_{k,new}]$

17: Update ${𝐐}_{k,old}\leftarrow [{𝐐}_{k,old};{𝐐}_{k,new}]$

**Updating ${𝐔}_{k,new}$.** We update ${𝐔}_{k,new}$ by setting $\frac{\partial ℒ}{\partial {𝐔}_{k,new}}=0$ and then rearranging the terms with the following lemma:

**Lemma 8.** *When fixing all the factor matrices except for* ${𝐔}_{k,new}$*, the following update for* ${𝐔}_{k,new}$ *minimizes* ${ℒ}_{OKG-CTF}$ *(Eq (*10*)):*

$${𝐔}_{k,new}\leftarrow \left({𝐓}_{k,new}𝐕{𝐒}_{k}+\lambda_{u}{𝐐}_{k,new}𝐇\right)\;\times {\left({𝐒}_{k}{𝐕}^{T}𝐕{𝐒}_{k}+\lambda_{u}𝐈\right)}^{-1}$$ (11)

□

*Proof*: See Appendix.

**Updating ${𝐒}_{k}$.** To update ${𝐒}_{k}$, we first transform ${𝐒}_{k}$ for *k* = 1...*K* into $𝐖\in {\mathbb{R}}^{K\times R}$ whose *k*-th row contains the diagonal elements of ${𝐒}_{k}$ (i.e., $𝐖(k,r)={𝐒}_{k}(r,r)$). Then, we update 𝐖(k,:) by setting $\frac{\partial ℒ}{\partial 𝐖(k,:)}=0$ and then rearrange the term based on the loss function (Eq (10)). We update **W** row by row with the following lemma:

**Lemma 9.** *When fixing all the factor matrices except for* 𝐖, *the following update for the k-th row of the factor matrix* 𝐖 *which corresponds to the diagonal elements of* ${𝐒}_{k}$ *minimizes* ${ℒ}_{OKG-CTF}$ *(Eq (*10*)):*

$$𝐖(k,:)\leftarrow (\lambda_{f}vec({𝐓}_{k,old}{)}^{T}(𝐕⊙{𝐔}_{k,old})+vec({𝐓}_{k,new}{)}^{T}(𝐕⊙{𝐔}_{k,new})\;+vec({𝐆}_{k}{)}^{T}(𝐑⊙{𝐌}_{k})+\lambda_{r}1^{T}(𝐑+{𝐃}_{k}^{-1}{𝐆}_{k}^{T}{𝐌}_{k}))\;\times (\lambda_{f}{𝐕}^{T}𝐕*{𝐔}_{k,old}^{T}{𝐔}_{k,old}+{𝐕}^{T}𝐕*{𝐔}_{k,new}^{T}{𝐔}_{k,new}\;+{𝐑}^{T}𝐑*{𝐌}_{k}^{T}{𝐌}_{k}+\lambda_{r}L𝐈+\lambda_{l}𝐈{)}^{-1}$$ (12)

*where* vec(·) *is a vectorization of a matrix.* ⊙ and * denote the Khatri-Rao product and the element-wise multiplication, respectively. $1\in {\mathbb{R}}^{L\times 1}$ *is a vector filled with ones.* □

*Proof*: See Appendix.

**Updating 𝐕.** We update 𝐕 by setting $\frac{\partial ℒ}{\partial 𝐕}=0$ and then rearranging the terms with the following lemma:

**Lemma 10.** *When fixing all the factor matrices except for* 𝐕*, the following update for* 𝐕 *minimizes* ${ℒ}_{OKG-CTF}$ *(Eq (*10*)):*

$$𝐕\leftarrow \left(\sum_{k=1}^{K}\left(\lambda_{f}{𝐓}_{k,old}^{T}{𝐔}_{k,old}{𝐒}_{k}+{𝐓}_{k,new}^{T}{𝐔}_{k,new}{𝐒}_{k}\right)\right)\;\times {\left(\lambda_{l}𝐈+\sum_{k=1}^{K}\left(\lambda_{f}{𝐒}_{k}{𝐔}_{k,old}^{T}{𝐔}_{k,old}{𝐒}_{k}+{𝐒}_{k}{𝐔}_{k,new}^{T}{𝐔}_{k,new}{𝐒}_{k}\right)\right)}^{-1}$$ (13)

□

*Proof*: See Appendix.

**Updating ${𝐐}_{k,new}$ and 𝐇.** We update ${𝐐}_{k,new}$ and 𝐇, which are factor matrices for the unique regularization, with the following lemma:

**Lemma 11.** *When fixing all the factor matrices except for* ${𝐐}_{k,new}$*, the following update for the matrix* ${𝐐}_{k,new}$ *minimizes the loss function (Eq (*10*)) by solving Orthogonal Procrustes problem [29] due to column-orthogonality:*

$${𝐐}_{k,new}\leftarrow {𝐙}_{k}{𝐏}_{k}^{T}$$ (14)

*where* ${𝐐}_{k,new}$ *is a column-orthogonal matrix (i.e.,* ${𝐐}_{k,new}^{T}{𝐐}_{k,new}=𝐈$)*, and* ${𝐙}_{k}$ *and* ${𝐏}_{k}^{T}$ *are left and right singular vector matrices of* ${𝐔}_{k,new}{𝐇}^{T}$*, respectively.* □

*Proof*: See Appendix.

**Lemma 12.** *When fixing all the factor matrices except for* 𝐇*, the following update for the factor matrix* 𝐇 *minimizes the loss function (Eq (*10*)):*

$$𝐇\leftarrow \frac{1}{K(\lambda_{f}+1)}\sum_{k=1}^{K}\left(\lambda_{f}{𝐐}_{k,old}^{T}{𝐔}_{k,old}+{𝐐}_{k,new}^{T}{𝐔}_{k,new}\right)$$ (15)

□

*Proof*: See Appendix.

**Updating ${𝐌}_{k}$ and 𝐑.** We update ${𝐌}_{k}$ by setting $\frac{\partial ℒ}{\partial {𝐌}_{k}}=0$ and $\frac{\partial ℒ}{\partial 𝐑}=0$, and then rearranging the terms with the following lemma:

**Lemma 13.** *The update rule for* ${𝐌}_{k}$ *to minimize* ${ℒ}_{OKG-CTF}$ *(Eq (*10*)) is identical to* ${𝐌}_{k}$ *defined in Lemma 6 for* ${ℒ}_{KG-CTF}$ *(Eq (*2*)).* □

**Lemma 14.** *The update rule for* 𝐑 *to minimize* ${ℒ}_{OKG-CTF}$ *(Eq (*10*)) is identical to* 𝐑 *defined in Lemma 7 for* ${ℒ}_{KG-CTF}$ *(Eq (*2*)).* □

We alternatively update factor matrices with our update procedure in Lemmas 8 to 14. Each update is the exact closed-form ALS solution of a quadratic subproblem with all other factors fixed, which ensures monotonic decrease of the objective and convergence to a stationary local minimum.

### Time complexity of OKG-CTF

We provide the time complexity of OKG-CTF for updating the factor matrices.

**Theorem 2.** *The time complexity of OKG-CTF is given by*


$$𝒪(KR^{2}(J+L+R)+\sum_{k=1}^{K}(R(J+R)I_{k,new}+R(L+R)N_{k}+N_{k}^{2}R+N_{k}^{3})).$$

□

*Proof*: See Appendix.

Theorem 2 establishes that OKG-CTF’s time cost is linear in the time length *I*_*k*,*new*_ of the newly arrived data, with $I_{k,new}\ll I_{k}$ as the tensor grows. This leads to significantly reduced computational cost, especially in long streaming sequences.

## Experiments

We conduct experiments to explore the following research questions:

Q1. **Offline performance.** How accurately does KG-CTF predict missing values in real-world irregular tensors?
Q2. **Online streaming performance.** How efficiently and accurately does OKG-CTF update factor matrices when new data are added to existing slice matrices?
Q3. **Ablation study.** How do the relational regularization and the accelerated update method contribute to the overall performance of KG-CTF and OKG-CTF?
Q4. **Hyperparameter sensitivity.** How much do the hyperparameters affect the performance of KG-CTF and OKG-CTF?

### Experimental settings

We provide a detailed overview of our experimental setup, including datasets, baselines, task descriptions, evaluation metrics, and hyperparameters.

**Datasets.** We conduct experiments on six real-world datasets, as summarized in Tables 2 and 3. Each dataset comprises a temporal irregular tensor paired with a corresponding knowledge graph tensor. These datasets cover stock markets from the United States (S&P500, NYSE, NASDAQ), South Korea, China, and Japan. For the stock datasets, each temporal irregular tensor consists of a collection of matrices, where each matrix corresponds to an individual stock. Each slice matrix follows a (date, feature) format, with features categorized into two groups: (1) six fundamental features, including opening price, closing price, highest price, lowest price, adjusted closing price, and trading volume, and (2) 83 technical indicators derived from these fundamental features using the Technical Analysis library (https://technical-analysis-library-in-python.readthedocs.io/en/latest/). For the knowledge graph datasets, we construct triple-based knowledge graphs covering all stocks in the six datasets using the ICKG [30] model. The StockKG dataset contains a total of 89,822 entities, including 14,019 stock entities and 15 distinct types of relations. These datasets are represented as irregular tensors in the format (entity, relation, stock), where each slice matrix is structured as (entity, relation). Here, entities denote the set of associated entities for each stock, and the dataset comprises 15 distinct relation types.

**Table 2:** Description of real-world irregular tensor data. # of nnz indicates the number of nonzeros.

	Total Tensor Size				
**Dataset**	**Max Dim. *I***_***k***_	**Dim. *J***	**Dim. *K***	**# of nnz**	**Summary**
S&P500^1^	3,652	88	500	15M	Stock
NASDAQ^2^	10,779	88	1,564	32M	Stock
NYSE^2^	5,297	88	1,050	48M	Stock
Korea Stock^3^	6,173	6	1,181	37M	Stock
China Stock^3^	4,167	6	4,550	80M	Stock
Japan Stock^3^	5,898	6	3,221	114M	Stock
StockKG^4^	78	15	13,458	119K	Knowledge graph

^1^ https://www.kaggle.com/datasets/camnugent/sandp500 ^2^ https://www.kaggle.com/datasets/paultimothymooney/stock-market-data ^3^ https://pypi.org/project/yfinance/ ^4^ https://xiaohui-victor-li.github.io/FinDKG/

**Table 3:** Description of real-world irregular tensor data in a streaming setting. Update cycle denotes the number of time steps between each update.

Dataset	Initial Tensor Size				Newly Arrived Data
	Max Dim. *I*_*k*_	Dim. *J*	Dim. *K*	Update Cycle	
S&P500	1,460	88	500	20 days	New rows of existing slice matrices
NASDAQ	888	88	561	20 days	
NYSE	1,472	88	757	20 days	
Korea Stock	1,696	6	801	20 days	
China Stock	1,304	6	1,252	20 days	
Japan Stock	1,712	6	2,426	20 days	

We preprocess the real-world datasets by applying normalization methods based on their specific characteristics. For the six stock datasets, we perform z-normalization on each *j*-th column 𝐗(:,j) of the slice matrix **X**. In contrast, the knowledge graph tensor, consisting of binary values (0 or 1), remains unchanged without any additional normalization.

**Competitors.** We compare KG-CTF against existing PARAFAC2 decomposition methods designed for irregular tensors:

- **PARAFAC2-ALS**: A standard PARAFAC2 decomposition approach utilizing alternating least squares. This method iteratively updates the target factor matrix while keeping all other factor matrices fixed.
- **ATOM** [15]: A PARAFAC2-based method tailored for handling missing values in temporal irregular tensors. ATOM introduces temporal smoothness regularization, ensuring gradual changes in temporal factor matrices over time.
- **CTF-ALS**: A Coupled Tensor Factorization (CTF) approach that employs ALS to jointly decompose multiple related tensors sharing a common axis. This method is designed to capture shared patterns across coupled tensors.
- **TASTE** [19]: A coupled matrix and tensor factorization framework that integrates both temporal and static features. It combines a non-negative PARAFAC2 model with non-negative matrix factorization to improve joint modeling capabilities.

We compare OKG-CTF with existing streaming PARAFAC2 decomposition methods in an online streaming setting:

- **PARAFAC2-ALS**: A baseline PARAFAC2 decomposition method to iteratively update the target factor matrix using alternating least squares.
- **SPADE** [31]: An efficient PARAFAC2 decomposition method designed to update new slice matrices.
- **DASH** [32]: An advanced PARAFAC2 decomposition method capable of efficiently handling both new slice matrices and new rows in existing slice matrices.
- **CTF-ALS**: A Coupled Tensor Factorization (CTF) method using ALS to jointly factorize multiple related tensors sharing a common axis, learning shared patterns across coupled tensors.

**Task.** To evaluate the performance of KG-CTF and OKG-CTF, we perform a missing value prediction task on temporal irregular tensor data. We randomly divide the data into training and test entries with the following ratios: (90%,10%),(80%,20%), and (70%,30%).

**Metric.** We evaluate the model performance using the Root Mean Squared Error (RMSE) computed as $\sqrt{\frac{\sum_{(i,j,k)\in \Omega }\left(𝐗(i,j,k)-\hat{𝐗}(i,j,k\right){)}^{2}}{\left|\Omega \right|}}$, where Ω represents the set of test entries in the input tensor **X**, and $\hat{𝐗}$ denotes the reconstructed tensor obtained from the learned factor matrices. A lower RMSE score indicates better tensor decomposition accuracy.

**Hyperparameters.** The hyperparameters for KG-CTF and OKG-CTF include the target rank *R* and the regularization strengths $\lambda_{u},\lambda_{l}$, and $\lambda_{r}$, along with the momentum coefficient *β*. We set the hyperparameters as follows: the target rank *R* to 5 for the China, Japan, and Korea datasets, and to 12 for the S&P500, NYSE, and NASDAQ datasets; the regularization strengths $\lambda_{u}$, $\lambda_{l}$, $\lambda_{r}$, and $\lambda_{f}$ to 10, 1, 0.01, and 0.1, respectively; and the momentum coefficient *β* to 0.5.

### Offline performance (Q1)

We compare the performance of KG-CTF with baseline models for the task of missing value prediction at test ratios ranging from 10% to 30%. According to Table 4, KG-CTF consistently achieves lower error rates across most datasets. For instance, in the Japan Stock dataset, KG-CTF shows an error rate approximately 1.64× lower than that of PARAFAC2-ALS at 10% test ratio and about 1.52× lower at 30% test ratio. Traditional methods reveal their limitations as they focus primarily on learning dynamic features while neglecting static features. In the NASDAQ, S&P500, and Korea Stock datasets, however, the performance gaps are less pronounced since KG coverage is skewed—a few stocks are linked to many entities while most have very few—so KG signals enhance a limited subset of stocks, reducing the overall benefit of coupling. In contrast, NYSE, China, and Japan Stock datasets exhibit more balanced entity coverage, allowing KG signals to be utilized more uniformly across stocks and resulting in more consistent performance improvements. Additionally, as the test ratio increases from 10% to 30%, the reliance of competing models on dynamic features causes their performance to degrade more significantly, further widening the gap with KG-CTF.

**Table 4:** Performance for missing value prediction. Note that bold and underlined fonts indicate the lowest and second-lowest errors, respectively. KG-CTF outperforms the competitors across all datasets and missing value ratios.

Model	NASDAQ	NYSE	S&P500	Korea Stock	China Stock	Japan Stock
**Missing value prediction - test ratio: 10%**						
PARAFAC2-ALS	0.4553 ± 0.0103	0.4092 ± 0.0114	0.5574 ± 0.0161	0.3495 ± 0.0202	0.3977 ± 0.0045	0.4337 ± 0.0143
ATOM	0.4127 ± 0.0011	0.3422 ± 0.0242	0.5232 ± 0.0353	**0.3082 ± 0.0072**	0.3592 ± 0.0102	0.3803 ± 0.0113
CTF-ALS	0.4337 ± 0.0115	0.3415 ± 0.0215	0.5340 ± 0.0146	0.3210 ± 0.0120	0.3719 ± 0.0072	0.3515 ± 0.0103
TASTE	0.4253 ± 0.0035	0.2601 ± 0.0023	0.5213 ± 0.0102	0.3328 ± 0.0042	0.3318 ± 0.0017	0.3255 ± 0.0052
KG-CTF	**0.4033 ± 0.0029**	**0.2489 ± 0.0026**	**0.5093 ± 0.0012**	0.3140 ± 0.0012	**0.3179 ± 0.0023**	**0.2652 ± 0.0013**
**Missing value prediction - test ratio: 30%**						
PARAFAC2-ALS	0.4951 ± 0.0103	0.4231 ± 0.0311	0.5894 ± 0.0132	0.3902 ± 0.0014	0.4230 ± 0.0014	0.4519 ± 0.0201
ATOM	**0.4234 ± 0.0025**	0.3521 ± 0.0053	0.5683 ± 0.0032	0.3447 ± 0.0081	0.3816 ± 0.0112	0.4042 ± 0.0121
CTF-ALS	0.4653 ± 0.0103	0.3667 ± 0.0231	0.5545 ± 0.0012	0.3512 ± 0.0063	0.4165 ± 0.0249	0.3767 ± 0.0053
TASTE	0.4434 ± 0.0082	0.2890 ± 0.0105	0.5663 ± 0.0101	0.3541 ± 0.0021	0.3880 ± 0.0013	0.3503 ± 0.0013
KG-CTF	0.4287 ± 0.0115	**0.2729 ± 0.0012**	**0.5368 ± 0.0081**	**0.3432 ± 0.0102**	**0.3516 ± 0.0058**	**0.2981 ± 0.0076**

### Online performance for newly arrived data (Q2)

We evaluate the performance of OKG-CTF with baselines in terms of local errors and efficiency.

**Local Error.** We evaluate OKG-CTF’s performance by analyzing local errors, measuring the mean and standard deviation across all updates. Table 5 shows that OKG-CTF consistently achieves much lower local errors than competing models. For example, in the S&P500 dataset, OKG-CTF performs up to 1.17× better than existing models. This is because OKG-CTF learns both dynamic and static features by incorporating auxiliary information with each new data arrival, resulting in more accurate factor matrices.

**Table 5:** Performance for missing value prediction in an online setting. Note that bold and underlined fonts indicate the lowest and second-lowest errors. OKG-CTF outperforms the competitors across all datasets and missing value ratios.

Local errors - test ratio: 20%						
**Model**	**NASDAQ**	**NYSE**	**S&P500**	**Korea Stock**	**China Stock**	**Japan Stock**
PARAFAC2-ALS	0.4511 ± 0.0044	0.4944 ± 0.0054	0.3844 ± 0.0012	0.3399 ± 0.0110	0.4096 ± 0.0065	0.3505 ± 0.0028
SPADE	0.4509 ± 0.0042	0.5022 ± 0.0139	0.3847 ± 0.0024	0.3399 ± 0.0089	0.4011 ± 0.0024	0.3499 ± 0.0031
DASH	0.4584 ± 0.0039	0.4833 ± 0.0051	0.3960 ± 0.0014	0.3341 ± 0.0091	0.3821 ± 0.0016	0.3572 ± 0.0033
CTF-ALS	0.4514 ± 0.0044	0.5021 ± 0.0050	0.3852 ± 0.0310	0.3396 ± 0.0090	0.4287 ± 0.0046	0.3641 ± 0.0029
OKG-CTF	**0.4014 ± 0.0099**	**0.4513 ± 0.0084**	**0.3384 ± 0.0017**	**0.3148 ± 0.0079**	**0.3735 ± 0.0079**	**0.3201 ± 0.0111**

**Fig 5:**
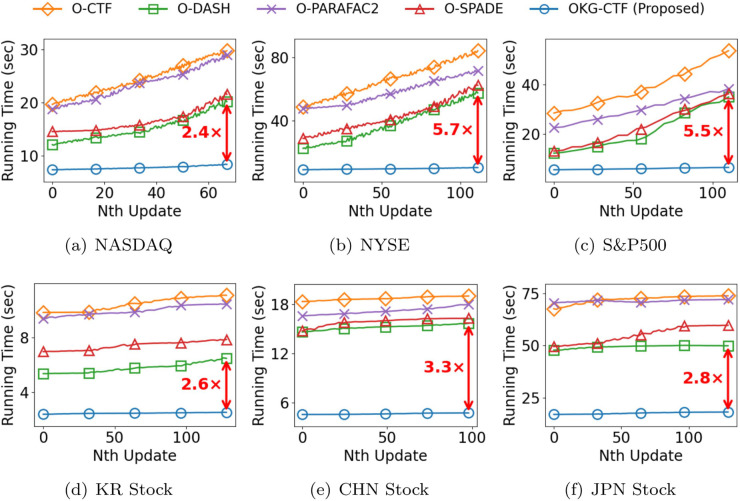
Running time of OKG-CTF and competitors for a new tensor on real-world datasets.

**Efficiency.** We evaluate the performance of OKG-CTF compared to competing models in a streaming setting. In Fig 5, the running time for the *N*-th update represents the update time for the *N*-th newly arrived data, rather than cumulative time. Fig 5 presents the results for all datasets in a streaming setting where new rows of existing slice matrices arrive. OKG-CTF demonstrates superior performance, achieving up to 5.7 × faster speeds compared to traditional static PARAFAC2 decomposition methods and streaming PARAFAC2 decomposition methods. Notably, in the NYSE and S&P500 datasets, Fig 5(b) and Fig 5(c) show that OKG-CTF achieves faster updates compared to competing models. This shows that OKG-CTF maintains competitive speed performance, while combining static and dynamic features to learn richer information.

**Fig 6:**
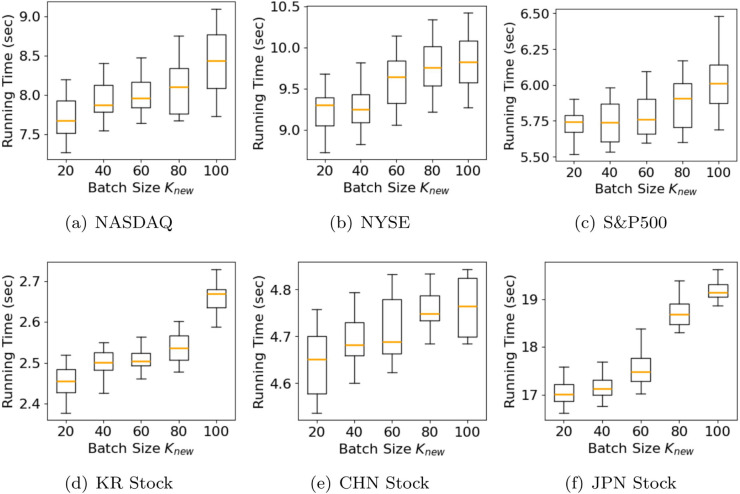
Scalability of OKG-CTF with respect to five update cycles: [20,40,60,80,100]. The size of new rows of existing slice matrices is linearly proportional to an update cycle.

**Scalability for newly arrived data size.** We evaluate the scalability of OKG-CTF with respect to the size of a new incoming tensor by measuring its running time. We measure execution times across five update cycles [20, 40, 60, 80, 100]. The length of new rows added to existing slice matrices increases linearly with the update cycle. As shown in Fig 6, the results are presented using box plots: the orange line marks the median, the box covers the upper (*Q*3) and lower (*Q*1) quartiles, and the horizontal lines represent 2.5×Q3−1.5×Q1 and 2.5×Q1−1.5×Q3. Fig 6 clearly shows that, in the setting where only new rows are added to existing slice matrices, the execution time of OKG-CTF scales linearly with the update cycle.

### Ablation study (Q3)

We conduct an ablation study to evaluate how relationship learning from the knowledge graph and accelerated learning via momentum updates affect prediction accuracy for both KG-CTF and OKG-CTF. Table 6 provides the global error results for KG-CTF under 20% missing-value test ratio, while Table 7 reports the local error results for OKG-CTF under 30% test ratio. For each method, we consider three reduced variants: (i) KG-CTF-R and OKG-CTF-R, which omit the relational regularization derived from the knowledge graph; (ii) KG-CTF-M and OKG-CTF-M, which exclude the momentum updates; (iii) KG-CTF-RM and OKG-CTF-RM, which remove both components and thus resemble traditional CTF-ALS. As shown in Tables 6 and 7, KG-CTF and OKG-CTF consistently show the lowest prediction errors, confirming that incorporating both relational information and momentum updates leads to superior performance. Even though KG-CTF-R and OKG-CTF-R lose some predictive accuracy by excluding knowledge graph relationships, they still outperform their respective -RM variants. Likewise, KG-CTF-M and OKG-CTF-M underscore the benefit of momentum updates, since their performance degrades relative to the full models. These results collectively demonstrate that our models effectively exploit both static relational structure and dynamic temporal information, allowing them to learn more accurate factor matrices for irregular time-evolving tensors.

**Table 6:** Ablation study of KG-CTF. -R and -M indicate the elimination of relational regularization and no momentum update, respectively. Bold and underlined fonts indicate the lowest and second-lowest errors. Note that both the relational regularization and momentum update contribute to improving the prediction accuracy.

Missing value prediction - test ratio: 20%						
**Model**	**NASDAQ**	**NYSE**	**S&P500**	**Korea Stock**	**China Stock**	**Japan Stock**
KG-CTF- RM	0.4697 ± 0.0093	0.3523 ± 0.0012	0.5639 ± 0.0021	0.3452 ± 0.0034	0.3923 ± 0.0021	0.3652 ± 0.0161
KG-CTF- M	0.4562 ± 0.0017	0.3524 ± 0.0080	0.5425 ± 0.0037	0.3272 ± 0.0102	0.3773 ± 0.0047	0.3585 ± 0.0022
KG-CTF- R	0.4382 ± 0.0013	0.2750 ± 0.0120	0.5390 ± 0.0024	**0.3001 ± 0.0048**	0.3459 ± 0.0021	0.3142 ± 0.0073
KG-CTF	**0.4126 ± 0.0075**	**0.2672 ± 0.0023**	**0.5368 ± 0.0102**	0.3204 ± 0.0102	**0.3341 ± 0.0024**	**0.2755 ± 0.0023**

**Table 7:** Ablation study of OKG-CTF. -R and -M indicate the elimination of relational regularization and no momentum update, respectively. Bold and underlined fonts indicate the lowest and second-lowest errors. Note that both the relational regularization and momentum update contribute to improving the prediction accuracy.

Local errors - test ratio: 30%						
**Model**	**NASDAQ**	**NYSE**	**S&P500**	**Korea Stock**	**China Stock**	**Japan Stock**
OKG-CTF- RM	0.5204 ± 0.0052	0.5302 ± 0.0125	0.4286 ± 0.0093	0.4836 ± 0.0049	0.4786 ± 0.0054	0.5100 ± 0.0014
OKG-CTF- M	0.5017 ± 0.0030	**0.4984 ± 0.0021**	0.4089 ± 0.0026	0.4648 ± 0.0028	0.4561 ± 0.0022	0.5104 ± 0.0048
OKG-CTF- R	0.4977 ± 0.0022	0.5052 ± 0.0033	0.3947 ± 0.0137	0.4143 ± 0.0067	0.4222 ± 0.0048	0.4562 ± 0.0029
OKG-CTF	**0.4857 ± 0.0021**	0.5046 ± 0.0027	**0.3911 ± 0.0094**	**0.4098 ± 0.0029**	**0.4173 ± 0.0041**	**0.4365 ± 0.0081**

### Hyperparameter sensitivity (Q4)

**Fig 7:**
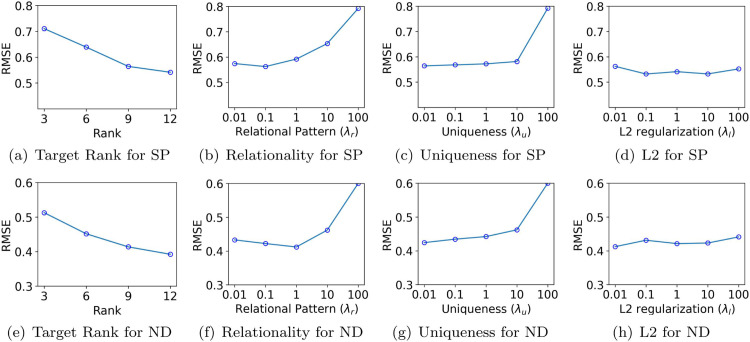
Hyperparameter sensitivity of KG-CTF in terms of Root Mean Squared error on S&P500 and NASDAQ datasets. Note that SP stands for S&P500 and ND stands for NASDAQ.

**Fig 8:**
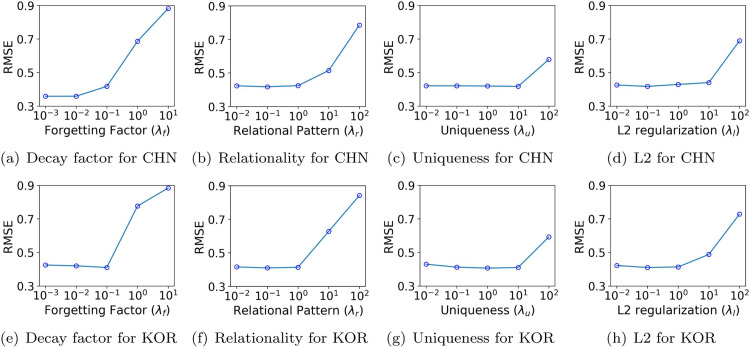
Hyperparameter sensitivity of OKG-CTF in terms of Root Mean Squared error on China Stock and Korea Stock datasets. Note that CHN stands for China Stock and KOR stands for Korea Stock.

We analyze the hyperparameter sensitivity of KG-CTF and OKG-CTF by measuring prediction errors across various key hyperparameters, including target rank, relational regularization, uniqueness, L2 regularization, forgetting factor, and momentum coefficient. Our experiments test four target rank values *R* of [3, 6, 9, 12], five relational regularization hyperparameters $\lambda_{r}$ of [0.01, 0.1, 1, 10, 100], five uniqueness hyperparameters $\lambda_{u}$ of [0.01, 0.1, 1, 10, 100], five L2 regularization hyperparameters $\lambda_{l}$ of [0.01, 0.1, 1, 10, 100], five forgetting factor hyperparameters $\lambda_{f}$ of [0.001, 0.01, 0.1, 1, 10], and five momentum coefficients *β* of [0.0, 0.3, 0.5, 0.7, 0.9]. We use the S&P500 (SP) and NASDAQ (ND) datasets to illustrate KG-CTF’s performance (see Fig 7), and the China Stock (CHN) and Korea Stock (KOR) datasets to examine OKG-CTF (see Fig 8).

**Rank.** We evaluate KG-CTF’s performance on S&P500 and NASDAQ datasets across four target rank values (3, 6, 9, 12). As shown in Fig 7(a) and 7(e), the prediction errors steadily decrease as the target rank increases, reaching their lowest at the highest rank *R* = 12. These results show that larger rank values consistently yield lower prediction errors, confirming that a higher rank helps capture more complex patterns in the data and improves accuracy.

**Relational pattern.** Both KG-CTF and OKG-CTF incorporate relational regularization derived from a knowledge graph. For KG-CTF, Fig 7(b) and 7(f) illustrate the influence of learning relationships from the knowledge graph for the NASDAQ and S&P500 datasets. Specifically, for the NASDAQ dataset, $\lambda_{r}$ value of 1 achieves lower errors compared to smaller values such as 0.1 or 0.01. In contrast, the S&P500 dataset performs better with smaller $\lambda_{r}$ values. This difference arises because the NASDAQ dataset contains a larger number of entities, allowing the model to effectively leverage richer relational patterns. However, excessively large values of $\lambda_{r}$ can lead to overfitting, particularly on datasets with fewer entities like S&P500, causing the model to learn noise rather than meaningful relational structures. A similar pattern appears for OKG-CTF in Fig 8(b) and 8(f). On both the China and Korea Stock datasets, moderate value of $\lambda_{r}$ (around 1) produces optimal performance, confirming the importance of appropriately tuning relational regularization to avoid overfitting.

**Uniqueness.** We assess the impact of uniqueness regularization on the performance of KG-CTF and OKG-CTF. In both KG-CTF (Fig 7(c), 7(g)) and OKG-CTF (Fig 8(c), 8(g)), we observe that an excessively large $\lambda_{u}$ degrades performance. Although moderate $\lambda_{u}$ helps improve the interpretability of the factor matrices, excessive regularization negatively affects the overall performance.

**L2.** We examine how L2 regularization affects prediction performance for both KG-CTF and OKG-CTF. As shown in Fig 7(d) and 7(h) (for KG-CTF), the error rates remain largely unchanged across different L2 regularization hyperparameter values. This stability arises from the normalized input tensors, which align the scales of the factor matrices, as well as from the abundant data in the KG-CTF datasets, which support accurate factor updates even without strong regularization. In contrast, Fig 8(d), 8(h) (for OKG-CTF) reveal that larger $\lambda_{l}$ values clearly increase prediction errors. Because OKG-CTF operates on newly arrived smaller datasets, excessive L2 regularization overly constrains the model, shrinking parameter values prematurely and preventing it from capturing subtle, local patterns. Thus, while the large datasets in KG-CTF reduce sensitivity to L2 regularization, careful tuning of $\lambda_{l}$ is necessary for OKG-CTF to balance between preventing overfitting and preserving essential information in smaller, more localized datasets.

**Forgetting factor.** OKG-CTF explicitly includes a forgetting factor $\lambda_{f}$ for time-evolving tensors. As illustrated in Fig 8(a) and 8(e) for the China and Korea stock datasets, excessively large values of $\lambda_{f}$ negatively impact the prediction accuracy by overly emphasizing outdated data, while very small values discard useful historical information too quickly. In Korea stock dataset, a moderate value ($\lambda_{f}=0.1$) achieves the best performance, balancing the short-term volatility and long-term trends inherent in stock data. Thus, selecting an intermediate value for $\lambda_{f}$ is crucial, enabling the model to dynamically adapt to recent data trends while preserving sufficient historical context for stable and accurate predictions.

**Fig 9:**
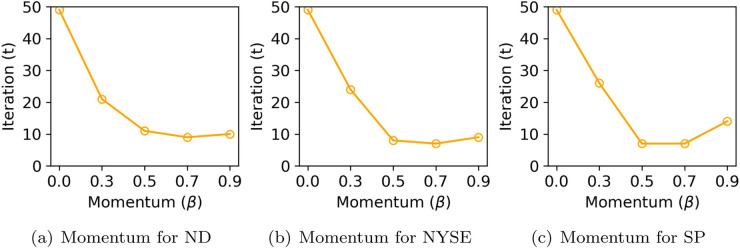
Convergence speed of KG-CTF across different momentum coefficients *β*, measured by the number of iterations (epochs). Note that ND and SP denote NASDAQ and S&P500, respectively.

**Fig 10:**
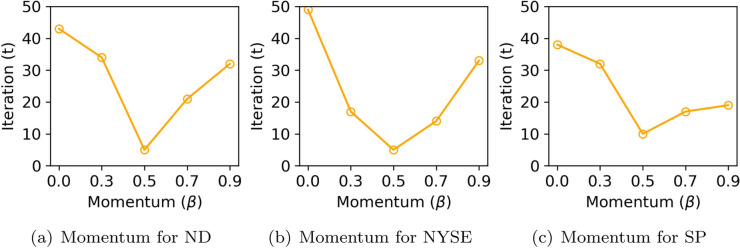
Convergence speed of OKG-CTF across different momentum coefficients *β*, measured by the number of iterations (epochs). Note that ND and SP denote NASDAQ and S&P500, respectively.

**Momentum coefficient.** We quantitatively analyze the impact of momentum-based updates on convergence speed in both KG-CTF and OKG-CTF. We measure convergence as the first iteration (epoch) at which the RMSE drops below a dataset-specific target threshold, with lower values indicating faster convergence. As shown in Fig 9, increasing the momentum coefficient *β* generally leads to faster convergence across the NASDAQ, NYSE, and S&P500 datasets. For example, in NASDAQ, the model with β=0.7 reaches the target RMSE within just 9 iterations, whereas the baseline without momentum (β=0.0) requires 49 iterations—indicating a 40-iteration improvement. This result highlights that the momentum mechanism becomes particularly effective in high-dimensional, large-scale datasets. However, in the S&P500 dataset, excessively large values (e.g., β≥0.9) slow down convergence or cause instability, indicating that overly strong momentum may harm training stability. Fig 10 shows similar trends in OKG-CTF. Across all datasets, β=0.5 consistently achieves the fastest convergence; for instance, NYSE reaches the target RMSE in only 5 iterations under this setting. By contrast, higher momentum values (β=0.7 or 0.9) result in slower convergence and degraded final performance. Overall, these results confirm that moderate momentum effectively accelerates convergence, whereas excessively large values can cause oscillations and suboptimal learning.

## Related work

We describe related works for existing tensor decomposition methods for irregular tensors, streaming tensors, and coupled tensor factorization approaches.

### Irregular tensor decomposition

Many studies [18,33] have introduced PARAFAC2 decomposition methods for analyzing irregular tensors. Unlike the traditional PARAFAC2-based ALS algorithm [22], RD-ALS [16] and DPar2 [14] apply preprocessing steps prior to factor matrix updates. ATOM [15] further improves the handling of temporal irregular sparse tensors, particularly those with missing values. However, they primarily focus on capturing dynamic features, while static features, which are equally crucial factors, are largely neglected. Integrating static features into the PARAFAC2 model often results in significant bias toward learning dynamic features. This limitation extends to other PARAFAC2-based approaches that do not explicitly incorporate side information. To address this, KG-CTF couples the temporal irregular tensor with the knowledge graph tensor by sharing a common axis, enabling the joint modeling of dynamic and static features while preserving the inherent relational structures among factor matrices.

### Streaming tensor decomposition

In online streaming settings, tensor decomposition methods [6,34,35] have been developed to efficiently update factor matrices as new data arrive. DAO-CP [9] adapts CP decomposition by detecting changes in tensor streams and selectively reusing or recomputing factor matrices. STF [7] incorporates attention-based temporal regularization to leverage past and future information for improved online learning. For irregular tensors, SPADE [31] updates factor matrices when new slices are added, while DASH [32] extends this by handling both new rows within existing slices and new slice matrices, enabling more flexible updates. However, existing methods focus on dynamic features, neglecting static features, which are equally important. A key limitation of online PARAFAC2 models is that they cannot incorporate static information in an online learning framework, as it remains unchanged over time and does not fit within their update mechanisms. While spectral regularization has been explored in neural networks and factorization models [36–38] to incorporate auxiliary structures, its use in online tensor factorization remains limited. Meanwhile, OKG-CTF effectively integrates dynamic and static features in online irregular tensor decomposition, enabling richer information learning while maintaining efficient factor matrix updates.

### Coupled tensor factorization

Previous studies [39–42] have proposed Coupled Matrix-Tensor Factorization (CMTF) methods to jointly decompose tensors and matrices. HaTen2 [43] and SCouT [44] extend CMTF by implementing distributed CP-based factorization within the MAPREDUCE framework. TASTE [19] introduces a coupled irregular tensor decomposition approach for EHR data, while C3APTION [45] builds on TASTE by integrating a (non-negative) PARAFAC2 model with a (non-negative) CP model. These methods leverage complementary information to achieve data fusion, enabling the extraction of richer information through tensor decomposition. However, relying on simple matrices to represent large-scale auxiliary information has limitations, particularly in capturing intricate relationships. In contrast, KG-CTF and OKG-CTF introduce novel data fusion methods that represent knowledge graph data as irregular tensors, effectively capturing relational structures within the factor matrices. KG-CTF [39] is our preliminary work on coupled tensor factorization, designed to jointly integrate temporal irregular tensors with knowledge graph tensors. In this work, we extend KG-CTF further to effectively handle streaming irregular tensors.

## Conclusion

In this paper, we propose KG-CTF and OKG-CTF, accurate coupled tensor factorization methods that extend the traditional PARAFAC2 approaches by incorporating a knowledge graph tensor. This integration enables the modeling of both dynamic and static characteristics within temporal irregular tensors in offline and online settings, respectively. Existing methods predominantly emphasize capturing dynamic temporal features while overlooking the intricate multi-dimensional relationships embedded in the data. To address these limitations, KG-CTF and OKG-CTF incorporate relational regularization to effectively capture relational structures within the knowledge graph. Additionally, a momentum-based update strategy is employed to accelerate the factor matrix updates. Our proposed methods achieve superior performance over existing PARAFAC2-based approaches by jointly learning dynamic and static features, leading to improved accuracy with minimal computational overhead. Extensive experimental evaluations confirm that both methods significantly reduce error rates, establishing them as robust solutions for complex temporal irregular tensor analysis. Future work includes extending KG-CTF and OKG-CTF to a broader range of datasets and further optimizing them to enhance scalability.

## Supporting information

S1 Text Detailed proofs for Lemmas 1 to 12 and Theorem 1 and 2. (PDF)
