# Supplementary material for: Offline and online coupled tensor factorization with knowledge graph
Source: PLoS One. 2025 Nov 12;20(11):e0336100. doi: 10.1371/journal.pone.0336100 (PMC12611170; doi:10.1371/journal.pone.0336100)
Supplement: S1 Text — (PDF) [file pone.0336100.s001.pdf]

Appendix

We provide proofs for Lemmas 1 to 12 and Theorems 1 and 2.

Proof of Lemma 1

Proof. We first compute $\frac{\partial \mathcal{L}}{\partial \mathbf{U}_k}$ as follows:

$$\frac{\partial \mathcal{L}}{\partial \mathbf{U}_k} = -2\left(\mathbf{T}_k - \mathbf{U}_k\mathbf{S}_k\mathbf{V}^T\right)\mathbf{V}\mathbf{S}_k + 2\lambda_u\left(\mathbf{U}_k - \mathbf{Q}_k\mathbf{H}\right)$$

Setting this to zero, we obtain Eq. (3).

□

Proof of Lemma 2

Proof. To obtain $\mathbf{W}(k,:)$, we first re-express the loss function:

$$\begin{aligned}\mathcal{L}_{\text{KG-CTF}} = \sum_{k=1}^{K}\Bigg(& \left\|\text{vec}(\mathbf{T}_k)^T - \mathbf{W}(k,:)\left(\mathbf{V}\odot\mathbf{U}_k\right)^T\right\|_F^2 + \lambda_u\left\|\mathbf{U}_k - \mathbf{Q}_k\mathbf{H}\right\|_F^2 \\ & + \left\|\text{vec}(\mathbf{G}_k)^T - \mathbf{W}(k,:)\left(\mathbf{R}\odot\mathbf{M}_k\right)^T\right\|_F^2 + \lambda_r\left\|\mathbf{1}\mathbf{W}(k,:) - \mathbf{R} - \mathbf{D}_k^{-1}\mathbf{G}_k^T\mathbf{M}_k\right\|_F^2 \\ & + \lambda_l\left(\|\mathbf{W}(k,:)\|_F^2 + \|\mathbf{M}_k\|_F^2\right)\Bigg) + \lambda_l\left(\|\mathbf{R}\|_F^2 + \|\mathbf{V}\|_F^2\right)\end{aligned}$$

Then, we compute $\frac{\partial \mathcal{L}}{\partial \mathbf{W}(k,:)}$ from the above function as follows:

$$\begin{aligned}\frac{\partial \mathcal{L}}{\partial \mathbf{W}(k,:)} = & -2\left(vec\left(\mathbf{T}_k\right)^T - \mathbf{W}(k,:)\left(\mathbf{V}\odot\mathbf{U}_k\right)^T\right)\left(\mathbf{V}\odot\mathbf{U}_k\right) \\ & -2\left(vec\left(\mathbf{G}_k\right)^T - \mathbf{W}(k,:)\left(\mathbf{R}\odot\mathbf{M}_k\right)^T\right)\left(\mathbf{R}\odot\mathbf{M}_k\right) \\ & + 2\lambda_r\mathbf{1}^T\left(\mathbf{1}\mathbf{W}(k,:) - \mathbf{R} - \mathbf{D}_k^{-1}\mathbf{G}_k^T\mathbf{M}_k\right) \\ & + 2\lambda_l\mathbf{W}(k,:)\end{aligned}$$

We set $\frac{\partial \mathcal{L}}{\partial \mathbf{W}(k,:)}$ to zero, and rearrange the equation for $\mathbf{W}(k,:)$, where $(\mathbf{A}\odot\mathbf{B})^T(\mathbf{A}\odot\mathbf{B})$ is equal to $(\mathbf{A}^T\mathbf{A}) * (\mathbf{B}^T\mathbf{B})$. Then, we obtain Eq. (4). □

Proof of Lemma 3

Proof. We first compute $\frac{\partial \mathcal{L}}{\partial \mathbf{V}}$ as follows:

$$\frac{\partial \mathcal{L}}{\partial \mathbf{V}} = -2\sum_{k=1}^{K}\left(\left(\mathbf{T}_k - \mathbf{U}_k\mathbf{S}_k\mathbf{V}^T\right)^T\mathbf{U}_k\mathbf{S}_k\right) + 2\lambda_l\mathbf{V}$$

Setting $\frac{\partial \mathcal{L}}{\partial \mathbf{V}}$ to zero, we obtain Eq. (5). □

Proof of Lemma 4

Proof. We minimize $\|\mathbf{U}_k - \mathbf{Q}_k\mathbf{H}\|_F^2$ with respect to $\mathbf{Q}_k$, where $\mathbf{Q}_k$ is a column-orthogonal matrix. This leads to the orthogonal Procrustes problem:

$$\begin{aligned}\underset{\mathbf{Q}k}{\text{minimize}} \quad & \|\mathbf{U}_k - \mathbf{Q}_k\mathbf{H}\|_F^2 \\ \text{subject to} \quad & \mathbf{Q}_k^T\mathbf{Q}_k = \mathbf{I}\end{aligned}$$

Note that this is equivalent to maximizing the following term:

$$tr(\mathbf{Q}_k^T \mathbf{U}_k \mathbf{H}^T) = \langle \mathbf{Z}_k^T \mathbf{Q}_k \mathbf{P}_k, \mathbf{\Sigma}_k \rangle_F$$

where tr and $\langle \cdot, \cdot \rangle_F$ denote the trace of a matrix and the Frobenius inner product, respectively. $\mathbf{Z}_k \mathbf{\Sigma}_k \mathbf{P}_k^T$ represents the singular value decomposition (SVD) of $\mathbf{U}_k \mathbf{H}^T$, and $\langle \mathbf{Z}_k^T \mathbf{Q}_k \mathbf{P}_k, \mathbf{\Sigma}_k \rangle_F$ is maximized when $\mathbf{Z}_k^T \mathbf{Q}_k \mathbf{P}_k = \mathbf{I}$, since $\mathbf{Z}_k$ and $\mathbf{Q}_k \mathbf{P}_k$ are all column-orthogonal matrices. Therefore, $\mathbf{Q}_k$ is updated as $\mathbf{Z}_k \mathbf{P}_k^T$ as in Eq. (6). □

Proof of Lemma 5

Proof. We compute $\frac{\partial \mathcal{L}}{\partial \mathbf{H}}$ as follows:

$$\frac{\partial \mathcal{L}}{\partial \mathbf{H}} = -2\lambda_u \sum_{k=1}^{K} \left(\mathbf{Q}_k^T (\mathbf{U}_k - \mathbf{Q}_k \mathbf{H}) \right)$$

We set $\frac{\partial \mathcal{L}}{\partial \mathbf{H}}$ to zero, arrange the equation for $\mathbf{H}$, and obtain Eq. (7). Note that $\mathbf{Q}_k$ is a column-orthogonal matrix. □

Proof of Lemma 6

Proof. We first compute $\partial \mathcal{L} / \partial \mathbf{M}_k$ as follows:

$$\begin{aligned} \frac{\partial \mathcal{L}}{\partial \mathbf{M}_k} = & -2 \left(\mathbf{G}_k \mathbf{R} \mathbf{S}_k - \mathbf{M}_k \mathbf{S}_k \mathbf{R}^T \mathbf{R} \mathbf{S}_k \right) \\ & - 2\lambda_r \left(\mathbf{G}_k \mathbf{D}_k^{-1} \right) \left(\mathbf{1}_H \mathbf{S}_k - \mathbf{R} - \mathbf{D}_k^{-1} \mathbf{G}_k^T \mathbf{M}_k \right) \\ & + 2\lambda_l \mathbf{M}_k \end{aligned}$$

Setting this to zero, we obtain Eq. (8). □

Proof of Lemma 7

Proof. We first compute $\frac{\partial \mathcal{L}}{\partial \mathbf{R}}$ as follows:

$$\begin{aligned} \frac{\partial \mathcal{L}}{\partial \mathbf{R}} = & -2 \sum_{k=1}^{K} \left(\left(\mathbf{G}_k - \mathbf{M}_k \mathbf{S}_k \mathbf{R}^T \right)^T \mathbf{M}_k \mathbf{S}_k \right) \\ & - 2\lambda_r \sum_{k=1}^{K} \left(\mathbf{1}_H \mathbf{S}_k - \mathbf{R} - \mathbf{D}_k^{-1} \mathbf{G}_k^T \mathbf{M}_k \right) + 2\lambda_l \mathbf{R} \end{aligned}$$

Setting $\frac{\partial \mathcal{L}}{\partial \mathbf{R}}$ to zero, we obtain Eq. (9). □

Proof of Theorem 1

Proof. First, consider the update of the mode-specific factors $\mathbf{U}_1, \ldots, \mathbf{U}_K$. For a fixed k the algorithm requires $\mathcal{O}\big(R(J+R)I_k\big)$ for matrix multiplication, $\mathcal{O}(JR^2)$ for the Gram matrix, and $\mathcal{O}(R^3)$ for matrix inversion. Summing over all K modes therefore yields the bound $\mathcal{O}\big(KR^2(J+R) \ + \ R(J+R)\sum_{k=1}^{K} I_k\big)$ for the collective cost of the U_k updates. The update of the shared factor $\mathbf{W}$ involves analogous products, which leads to the cost $\mathcal{O}\big(KR^2(J+L+R) \ + \ R(J+R)\sum_{k=1}^{K} I_k \ + \ R(L+R)\sum_{k=1}^{K} N_k\big)$. Next, updating the factor $\mathbf{V}$ costs $\mathcal{O}\big(KR^2J + R^3 + R(J+R)\sum_{k=1}^{K} I_k\big)$, and the auxiliary matrices $\mathbf{Q}_k$ and $\mathbf{H}$ are updated via $\mathcal{O}\big(R^2 \sum_{k=1}^{K} I_k\big)$ costs since the SVD of $\mathbf{U}_k \mathbf{H}^T$ requires $\mathcal{O}(I_k R^2)$ computation provided that $R \ll I_k$ (a mild assumption in practice). Solving the Sylvester equation in Lemma 6 to update $\mathbf{M_k}$ for all k requires $\mathcal{O}\big(KR^2(R+L) + \sum_{k=1}^{K}(N_k R(L+R) + N_k^2 R + N_k^3)\big)$. Finally, the matrix $\mathbf{R}$ is updated by $\mathcal{O}\big(KR^2L + R^3 + R(L+R)\sum_{k=1}^{K} N_k\big)$ flops. Adding the all terms completes the proof. □

Proof of Lemma 8

Proof. We compute $\partial\mathcal{L}/\partial\mathbf{U}_{k,new}$ as follows:

$$\frac{\partial\mathcal{L}}{\partial\mathbf{U}_{k,new}} = -2\left(\mathbf{T}_{k,new} - \mathbf{U}_{k,new}\mathbf{S}_k\mathbf{V}^T\right)\mathbf{V}\mathbf{S}_k + 2\lambda_u\left(\mathbf{U}_{k,new} - \mathbf{Q}_{k,new}\mathbf{H}\right)$$

We set this to zero, arrange the equation for $\mathbf{U}_{k,new}$, and obtain Eq. (11). Note that $\mathbf{Q}_k$ is a column-orthogonal matrix. □

Proof of Lemma 9

Proof. To obtain $\mathbf{W}(k,:)$, we first re-express the loss function:

$$\begin{aligned}\mathcal{L}_{OKG-CTF} = \sum_{k=1}^{K}\Bigg(&\lambda_f\left\|\text{vec}(\mathbf{T}_{k,old})^T - \mathbf{W}(k,:)\left(\mathbf{V}\odot\mathbf{U}_{k,old}\right)^T\right\|_F^2 \\ &+ \left\|\text{vec}(\mathbf{T}_{k,new})^T - \mathbf{W}(k,:)\left(\mathbf{V}\odot\mathbf{U}_{k,new}\right)^T\right\|_F^2 \\ &+ \lambda_u\left(\lambda_f\left\|\mathbf{U}_{k,old} - \mathbf{Q}_{k,old}\mathbf{H}\right\|_F^2 + \left\|\mathbf{U}_{k,new} - \mathbf{Q}_{k,new}\mathbf{H}\right\|_F^2\right) \\ &+ \left\|\text{vec}(\mathbf{G}_k)^T - \mathbf{W}(k,:)\left(\mathbf{R}\odot\mathbf{M}_k\right)^T\right\|_F^2 + \lambda_r\left\|\mathbf{1}\mathbf{W}(k,:) - \mathbf{R} - \mathbf{D}_k^{-1}\mathbf{G}_k^T\mathbf{M}_k\right\|_F^2 \\ &+ \lambda_l\left(\|\mathbf{W}(k,:)\|_F^2 + \|\mathbf{M}_k\|_F^2\right)\Bigg) + \lambda_l\left(\|\mathbf{R}\|_F^2 + \|\mathbf{V}\|_F^2\right)\end{aligned}$$

We compute $\frac{\partial\mathcal{L}}{\partial\mathbf{W}(k,:)}$ as follows:

$$\begin{aligned}\frac{\partial\mathcal{L}}{\partial\mathbf{W}(k,:)} =& -2\lambda_f\left(vec\left(\mathbf{T}_{k,old}\right)^T - \mathbf{W}(k,:)\left(\mathbf{V}\odot\mathbf{U}_{k,old}\right)^T\right)\left(\mathbf{V}\odot\mathbf{U}_{k,old}\right) \\ &- 2\left(vec\left(\mathbf{T}_{k,new}\right)^T - \mathbf{W}(k,:)\left(\mathbf{V}\odot\mathbf{U}_{k,new}\right)^T\right)\left(\mathbf{V}\odot\mathbf{U}_{k,new}\right) \\ &- 2\left(vec\left(\mathbf{G}_k\right)^T - \mathbf{W}(k,:)\left(\mathbf{R}\odot\mathbf{M}_k\right)^T\right)\left(\mathbf{R}\odot\mathbf{M}_k\right) \\ &+ 2\lambda_r\mathbf{1}^T\left(\mathbf{1}\mathbf{W}(k,:) - \mathbf{R} - \mathbf{D}_k^{-1}\mathbf{G}_k^T\mathbf{M}_k\right) \\ &+ 2\lambda_l\mathbf{W}(k,:)\end{aligned}$$

We set $\frac{\partial\mathcal{L}}{\partial\mathbf{W}(k,:)}$ to zero, and rearrange the equation for $\mathbf{W}(k,:)$, where $\left(\mathbf{A}\odot\mathbf{B}\right)^T\left(\mathbf{A}\odot\mathbf{B}\right)$ is equal to $(\mathbf{A}^T\mathbf{A}) * (\mathbf{B}^T\mathbf{B})$. Then, we obtain Eq. (12). □

Proof of Lemma 10

Proof. We first compute $\frac{\partial\mathcal{L}}{\partial\mathbf{V}}$ as follows:

$$\begin{aligned}\frac{\partial\mathcal{L}}{\partial\mathbf{V}} =& -2\lambda_f\sum_{k=1}^{K}\left(\left(\mathbf{T}_{k,old} - \mathbf{U}_{k,old}\mathbf{S}_k\mathbf{V}^T\right)^T\mathbf{U}_{k,old}\mathbf{S}_k\right) \\ &- 2\sum_{k=1}^{K}\left(\left(\mathbf{T}_{k,new} - \mathbf{U}_{k,new}\mathbf{S}_k\mathbf{V}^T\right)^T\mathbf{U}_{k,new}\mathbf{S}_k\right) \\ &+ 2\lambda_l\mathbf{V}\end{aligned}$$

Setting $\frac{\partial\mathcal{L}}{\partial\mathbf{V}}$ to zero, we obtain Eq. (13). □

Proof of Lemma 11

Proof. We minimize $\|\mathbf{U}_{k,new} - \mathbf{Q}_{k,new}\mathbf{H}\|_F^2$ with respect to $\mathbf{Q}_{k,new}$, maximizing the following term:

$$tr(\mathbf{Q}_{k,new}^T\mathbf{U}_{k,new}\mathbf{H}^T) = \langle\mathbf{Z}_k^T\mathbf{Q}_{k,new}\mathbf{P}_k, \mathbf{\Sigma}_k\rangle_F$$

where tr and $\langle\cdot\rangle_F$ denote the trace of a matrix and the Frobenius inner product, respectively. $\mathbf{Z}_k\mathbf{\Sigma}_k\mathbf{P}_k^T$ represents the singular value decomposition (SVD) of $\mathbf{U}_{k,new}\mathbf{H}^T$, and $\langle\mathbf{Z}_k^T\mathbf{Q}_{k,new}\mathbf{P}_k, \mathbf{\Sigma}_k\rangle_F$ is maximized when $\mathbf{Z}_k^T\mathbf{Q}_{k,new}\mathbf{P}_k = \mathbf{I}$, since $\mathbf{Z}_k$ and $\mathbf{Q}_{k,new}\mathbf{P}_k$ are all column-orthogonal matrices. Therefore, $\mathbf{Q}_{k,new}$ is updated as $\mathbf{Z}_k\mathbf{P}_k^T$ as in Eq. (14). □

Proof of Lemma 12

Proof. We compute $\frac{\partial\mathcal{L}}{\partial\mathbf{H}}$ as follows:

$$\frac{\partial\mathcal{L}}{\partial\mathbf{H}} = -2\lambda_u \sum_{k=1}^{K} \Big(\lambda_f \mathbf{Q}_{k,old}^T(\mathbf{U}_{k,old} - \mathbf{Q}_{k,old}\mathbf{H}) + \mathbf{Q}_{k,new}^T(\mathbf{U}_{k,new} - \mathbf{Q}_{k,new}\mathbf{H})\Big)$$

We set $\frac{\partial\mathcal{L}}{\partial\mathbf{H}}$ to zero, arrange the equation for $\mathbf{H}$, and obtain Eq. (15). Note that $\mathbf{Q}_{k,new}$ is a column-orthogonal matrix. □

Proof of Theorem 2

Proof. Consider that each slice k in the streaming setting gains only $\mathbf{T}_{k,new}$ of size $I_{k,new}$. Updating $\mathbf{U}_{k,new}$ costs $\mathcal{O}\big(R(J+R)I_{k,new} + JR^2 + R^3\big)$ per slice, thus summing to $\mathcal{O}\big(KR^2(J+R) + R(J+R)\sum_{k=1}^{K} I_{k,new}\big)$ overall. Next, the shared factor $\mathbf{W}$ is split into old- and new-data terms, giving $\mathcal{O}\big(KR^2(J+L+R) + R(J+R)\sum_{k=1}^{K} I_{k,new} + R(L+R)\sum_{k=1}^{K} N_k\big)$, while updating $\mathbf{V}$ yields $\mathcal{O}\big(KR^2J + R^3 + R(J+R)\sum_{k=1}^{K} I_{k,new}\big)$. Then, the auxiliary matrices $\mathbf{Q}_{k,new}$ and $\mathbf{H}$ are updated via $\mathcal{O}\big(R^2\sum_{k=1}^{K} I_{k,new}\big)$ costs since the SVD of $\mathbf{U}_{k,new}\mathbf{H}^T$ requires $\mathcal{O}(I_{k,new}R^2)$ computation provided that $R \ll I_{k,new}$ (a mild assumption in practice). To update the entity factor $\mathbf{M}_k$, we solve a Sylvester equation (Lemma 13), requiring $\mathcal{O}\big(KR^2(R+L) + \sum_{k=1}^{K}(R(L+R)N_k + N_k^2R + N_k^3)\big)$. Finally, the matrix $\mathbf{R}$ is updated in $\mathcal{O}\big(KR^2L + R^3 + R(L+R)\sum_{k=1}^{K} N_k\big)$ time. Adding the terms completes the proof. □
